# Caspase-3/NLRP3 signaling in the mesenchymal stromal niche regulates myeloid-biased hematopoiesis

**DOI:** 10.1186/s13287-021-02640-y

**Published:** 2021-11-20

**Authors:** Jing Zhang, Yaozhen Chen, Dandan Yin, Fan Feng, Qunxing An, Zhixin Liu, Ning An, Jinmei Xu, Jing Yi, Shunli Gu, Wen Yin, Yazhou Wang, Xingbin Hu

**Affiliations:** 1 Department of Transfusion Medicine, Xijing Hospital, Fourth Military Medical University, Xi’an, 710032 Shaanxi China; 2 Department of Hematology, Tangdu Hospital, Fourth Military Medical University, Xi’an, Shaanxi China; 3 Division of Digestive Surgery, Xijing Hospital of Digestive Diseases, Fourth Military Medical University, Xi’an, Shaanxi China; 4 Institute of Neuroscience, Fourth Military Medical University, Xi’an, Shaanxi China

**Keywords:** Mesenchymal stromal cells, Hematopoiesis, Myeloid development, Caspase-3, NLRP3 inflammasome

## Abstract

**Background:**

The fate of hematopoietic stem cells (HSCs) is determined by a complex regulatory network that includes both intrinsic and extrinsic signals. In the past decades, many intrinsic key molecules of HSCs have been shown to control hematopoiesis homeostasis. Non-hematopoietic niche cells also contribute to the self-renewal, quiescence, and differentiation of HSCs. Mesenchymal stromal cells (MSCs) have been identified as important components of the niche. However, the regulatory role of MSCs in hematopoiesis has not been fully understood.

**Methods:**

Caspase-3 and NLRP3 gene knockout mice were generated respectively, and hematopoietic development was evaluated in the peripheral circulation and bone marrow by flow cytometry, colony formation assay, and bone marrow transplantation. Bone-associated MSCs (BA-MSCs) were then isolated from gene knockout mice, and the effect of Caspase-3/NLRP3 deficient BA-MSCs on hematopoiesis regulation was explored in vivo and ex vivo.

**Results:**

We report that Caspase-3 deficient mice exhibit increased myelopoiesis and an aberrant HSC pool. Ablation of Caspase-3 in BA-MSCs regulates myeloid lineage expansion by altering the expression of hematopoietic retention cytokines, including SCF and CXCL12. Interestingly, NLRP3 gene knockout mice share phenotypic similarities with Caspase-3 deficient mice. Additionally, we found that NLRP3 may play a role in myeloid development by affecting the cell cycle and apoptosis of hematopoietic progenitors.

**Conclusions:**

Our data demonstrate that the Caspase-3/NLRP3 signaling functions as an important regulator in physiological hematopoiesis, which provides new insights regarding niche signals that influence hematopoiesis regulation in the bone marrow.

**Supplementary Information:**

The online version contains supplementary material available at 10.1186/s13287-021-02640-y.

## Background

Hematopoiesis begins in the yolk sac during fetal development and arises from hemangioblasts. Later, the fetal liver becomes the main site of prenatal hematopoiesis. Hematopoietic stem cells (HSCs) then leave the fetal liver and relocate to the bone marrow (BM), where adult hematopoiesis mainly occurs [1, 2]. In 1978, the concept of a “niche” was proposed by Schofield [3], which provides a new perspective for the study of hematopoiesis regulation. It is believed that the BM microenvironment preserves the ability of maintenance, self-renewal, proliferation, and differentiation of HSCs [4, 5]. Both intrinsic and extrinsic regulatory signals control the fate of HSCs in vivo [6]. Notably, a growing number of non-hematopoietic niche cells have been identified in recent years, including osteoblasts, fibroblasts, endothelial cells, adipocytes, mesenchymal stromal cells (MSCs), etc. [7–9].

The hematopoietic niche not only provides support for the self-renewal and quiescence of HSCs but also shows biases in promoting the differentiation of HSCs into different lineages [10]. Kilroy et al*.* showed that adipose-derived stromal cells (ASCs) induced HSCs to differentiate into myeloid and lymphoid lineages in vitro, while the number of erythroid progenitors did not increase [11]. Moreover, our group previously demonstrated that Sca-1^+^ bone-associated MSCs (BA-MSCs) recovered erythropoiesis in a mouse model of cancer associated anemia [12]. Altogether, these results suggest that MSCs play a role in hematopoiesis regulation, but the underlying mechanisms have not been fully understood. Stem cell factor (SCF) and stromal cell-derived factor 1 (SDF-1/CXCL12) are cytokines that play key roles in the maintenance of HSCs and progenitors in the BM [13, 14]. Nestin^+^ niche cells were shown to have the ability to support the homing and retention of HSCs by expressing high levels of CXCL12 and SCF [15]. Similar to Nestin^+^ cells, CXCL12-abundant reticular (CAR) cells were also reported to support the survival and proliferation of lymphoid and erythroid progenitors, while ablation of CAR cells in mice was found to induce increased expression of myeloid transcription factor PU.1 in HSCs [16]. These findings suggest that MSCs have the potential to regulate myeloid development via cytokine secretion. However, the mechanism by which MSC-produced cytokines regulate hematopoiesis requires further exploration.

Caspases, which are a family of cysteine proteases, are known to play important roles in apoptosis, among which Caspase-3 is a major player [17, 18]. Studies have demonstrated that besides acting as an apoptotic executor, Caspase-3 also has several nonapoptotic functions [19]. For instance, it was revealed that Caspase-3 plays a role in the differentiation of skeletal muscle cells [20], megakaryocytes and erythroid maturation [21]. Scadden et al*.* reported that Caspase-3 deficiency in mice altered the cell cycle of hematopoietic progenitors and differentiation of B cells, by modulating the responsiveness of primitive hematopoietic cells to extracellular cytokines [22]. However, the role of Caspase-3 deficiency in the differentiation to a myeloid lineage has not been comprehensively studied. In addition, most studies have focused on the effect of Caspase-3 signaling on hematopoietic cells, while the role of Caspase-3 deficient niche cells (e.g., MSCs) in hematopoiesis regulation has been rarely explored.

The NLRP3 inflammasome recognizes pathogen-associated molecular patterns (PAMPs) or damage-associated molecular patterns (DAMPs), cleaves pro-Caspase-1 into Caspase-1, and induces the maturation and release of pro-inflammatory cytokines (e.g., IL-1β and IL-18) [23, 24]. Previously, we confirmed that activation of the NLRP3 inflammasome led to pyroptotic cell death in BA-MSCs [25], suggesting that the NLRP3 inflammasome also exists in MSCs. Moreover, it has been demonstrated that BAX/BAK signaling mediates Caspase-3/-7 activity, which subsequently triggers the NLRP3 inflammasome via potassium efflux in macrophages [26], indicating that there might be an interaction between Caspase-3 and NLRP3. Although they are key molecules in apoptosis and pyroptosis, the relationship between Casepase-3 and NLRP3, especially in MSCs, has not yet been elucidated. Caspase-3 was found to be abnormally expressed in leukemia patients [27], while the NLRP3 inflammasome has been shown to be associated with the incidence of blood disorders [28]. For instance, activation of the NLRP3 inflammasome induces myeloid proliferation in the BM [29]. These studies demonstrate that NLRP3 participates in hematopoiesis regulation, but the detailed mechanisms remain unclear.

In the present study, we obtained Caspase-3 and NLRP3 gene knockout mice and evaluated the hematopoietic development in the BM and peripheral circulation. Our results revealed that Caspase-3 deficiency in BA-MSCs regulates HSC and myeloid development, by altering the secretion of CXCL12 and SCF. Moreover, NLRP3 may also be involved in myeloid commitment by regulating the cell cycle and apoptosis of hematopoietic progenitors. Thus, our findings provide a better understanding of the function of niche signals in BM hematopoiesis.

## Methods

### Mice

Female C57BL/6 wild-type (WT) mice aged 8 to 12 weeks were obtained from the Animal Resources Center of the Fourth Military Medical University (Xi’an, China). Female C57BL/6 gene knockout mice (Caspase-3^−/−^ and NLRP3^−/−^) were maintained as previously described [30–33]. All mice were bred in a pathogen-free environment and were randomly assigned to the experiments. All animal experiments were approved by the Animal Care Committee of the Fourth Military Medical University, China.

### Reagents

The anti-mouse antibodies used for flow cytometry analyses were as follows: CD3-APC (#100236, Biolegend), CD11b-APC (#101212, Biolegend), Gr-1-APC (#108412, Biolegend), CD19-APC (#17-0193-80, eBioscience), B220-APC (#103212, Biolegend), Ter119-APC (#17-5921, eBioscience), c-kit-PE (#105807, Biolegend), Sca-1-PE-Cy7 (#558162, BD Biosciences), CD34-FITC (#343504, Biolegend), CD16/32-Percp-Cy5.5 (#560540, BD Biosciences), Flk2-BV421 (#135313, Biolegend), CD45.1-BV605 (#110737, Biolegend), CD45.2-APC-Cy7 (#109823, Biolegend), CD3-FITC (#100203, Biolegend), Gr-1-APC-Cy7 (#557661, BD Biosciences), CD11b-PE-Cy7 (#561098, BD Biosciences), CD19-BV605 (#563148, BD Biosciences), CD146-FITC (#134705, Biolegend), CD166-APC (#17-1661-80, eBioscience), CD45.1-Percp-Cy5.5 (#560580, BD Biosciences), and CD45.2-PE (#109807, Biolegend). The FITC Annexin V Apoptosis Detection Kit (#556547, BD Biosciences) was used to determine apoptosis levels. To assess the cell cycle, the Cell Cycle Kit was used with propidium staining (#C1052, Beyotime). The anti-mouse antibodies used for immunoblotting assays were as follows: Caspase-3 (#9662, Cell Signaling Technology), PARP (#9532, Cell Signaling Technology), NLRP3 (#AG-20B-0014, Adipogen), IL-1β (#12426, Cell Signaling Technology), ASC (#AG-25B-0006, Adipogen), GSDMDC1 (#sc-393656, Santa Cruz Biotechnology), alpha tubulin (#66031-1-1g, Proteintech), HRP goat anti-mouse IgG antibody (#EK010, Zhuangzhibio), and HRP goat anti-rabbit IgG antibody(#EK020, Zhuangzhibio).

### Cell isolation and culture

BM cells were isolated from the tibia and femur of 8- to 12-week-old mice. After removing the muscle and connective tissue and cutting off both ends of the bone, the bone marrow was flushed gently with phosphate-buffered saline (PBS) buffer using a syringe needle. BM cells were collected, and red blood cells were lysed using the Ammonium-Chloride-Potassium (ACK) lysis buffer. BM cells were then purified and sorted with magnetic beads using the EasySep Mouse Hematopoietic Progenitor Cell Isolation Kit (#19856, StemCell Technologies). The obtained hematopoietic stem/progenitor cells (HSPCs) were maintained in IMDM medium (Gibco) supplemented with 10% fetal bovine serum (FBS) at 37 °C. BA-MSCs were isolated and identified as previously reported [34] and cultured in MEM-alpha medium (Gibco) supplemented with 10% FBS at 37 °C. BA-MSCs passaged for a maximum of five times were used in all the experiments. Sca-1^+^ BA-MSCs were purified and sorted with magnetic beads using the EasySep Mouse SCA1 Positive Selection Kit (#18756, StemCell Technologies). For the co-culture of HSPCs and BA-MSCs, BA-MSCs (2 × 10^5^ cells/well) were seeded in a 6-well plate in MEM-alpha medium. After BA-MSC adhered in a monolayer, HSPCs were added to the supernatant in a ratio of 5:1 (1 × 10^6^ HSPCs/well). The cells from the upper layer were harvested for analysis or follow-up experiments after being co-cultured for 48 h. Recombinant Mouse SCF (#C775, Novoprotein) and CXCL12 (#C698, Novoprotein) were supplemented in MEM-alpha medium at the concentration of 50 ng/mL and 100 ng/mL respectively for the rescue experiment.

### Morphological analysis

The spleens of WT and Caspase-3^−/−^ mice were collected, and 10-μm frozen sections were prepared using a freezing microtome (Leica CM1860). For immunofluorescence staining, the spleen sections were fixed with 4% paraformaldehyde (PFA) and blocked with 5% bovine serum albumin (BSA). After staining with anti-CD11b (#101207, Biolegend) and DAPI (#D9542, Sigma–Aldrich), the samples were observed under a fluorescence microscope (Nikon). For hematoxylin–eosin (H&E) staining, the slides were stained with hematoxylin and eosin, respectively, according to the standard protocol, and observed under an optical microscope (Nikon) at 40 × and 200 × magnification.

### Flow cytometry analysis

Cells were harvested from mice or in vitro cultures and resuspended in PBS supplemented with 5% FBS and 0.4% NaN_3_. Red blood cells were lysed using the ACK lysis buffer, if necessary. The cells were then stained with specific antibodies at 4 °C for 20 min. After washing three times with PBS, stained cells were analyzed using the BD FACSCanto Plus flow cytometer (BD Biosystems) and the data were processed using the FlowJo software. Surface staining for lineage markers was performed using antibodies against CD3, CD11b (Mac-1α), Gr-1 (Ly-6G and Ly-6C), CD19, B220 (CD45R), and Ter119. To assess the primitive population, BM cells were stained with a cocktail of lineage antibodies, as described above, as well as c-kit (CD117)-PE, Sca-1 (Ly 6A/E)-PE-Cy7, CD34-FITC, CD16/32-Percp-Cy5.5, and Flk2 (CD135)-BV421. To evaluate the mature population, cells were stained with CD3-FITC, Gr-1-APC-Cy7, CD11b-PE-Cy7, B220-APC, and CD19-BV605. Anti-CD45.1 and anti-CD45.2 antibodies were used for congenic strain discrimination.

### Colony formation assay

HSPCs from WT and Caspase-3^−/−^ mice or from co-culture systems were isolated and sorted. Then, cells were seeded in 6-well plates at 500 cells/well and cultured in methylcellulose medium (MethoCult #3434, Stem Cell Technologies) supplemented with cytokines SCF, IL-3, IL-6, and EPO. Colonies were identified and counted after 7 and 14 days.

### Bone marrow transplantation assays

For BM transplantation, 3 × 10^6^ BM cells isolated from WT and Caspase-3^−/−^ mice (CD45.2) were injected into lethally irradiated (8.0 Gy) 8- to 12-week-old recipient mice (CD45.1). Engraftment efficiency in recipient mice was assessed by the donor contribution of CD45.2^+^ cells determined via flow cytometry analysis. For the competitive transplantation assay, 4 × 10^5^ HSPCs (CD45.1) collected from the co-culture system were mixed with 4 × 10^5^ WT helper cells (CD45.2) and injected into lethally irradiated (8.0 Gy) 8- to 12-week-old recipient mice (CD45.2). Engraftment efficiency in recipients was monitored by the donor contribution of CD45.1^+^ cells. Peripheral blood (PB) cells were analyzed by collecting 50 µL of blood from the ophthalmic vein at 4, 8, 12, and 16 weeks post-transplantation. BM subpopulation assessment was performed 16 weeks after the mice were sacrificed.

### Quantitative real-time PCR

Total RNA was extracted from BA-MSCs using an RNeasy RNA Isolation Mini Kit (#74104, Qiagen) and then reverse-transcribed into cDNA using the Sensiscript RT kit (#205211, Qiagen), according to the manufacturer’s instructions. Quantitative RT-PCR analysis was performed with the Tianlong Real-Time PCR System using the Fast Essential DNA Green Master Kit (#06402712001, Roche). Relative expression was determined by cycle-threshold (Ct) and normalized to the internal control GAPDH. For primer sequences, see Additional file 1: Table S1.

### ELISA

The concentrations of SCF and CXCL12 were measured using a Mouse SCF Quantikine ELISA Kit (#MCK00, R&D Systems) and Mouse CXCL12/SDF-1 alpha Quantikine ELISA Kit (#MCX120, R&D Systems), according to the manufacturer’s instructions. For ELISA experiments, cells were seeded at 2 × 10^5^ cells/well in 6-well plates and cultured for 7 days, after which the supernatants were collected for analysis.

### Western Blot

Cells were harvested and lysed using the RIPA reagent (#P0013C, Beyotime). Protein concentrations were measured using a BCA Protein Assay Kit (#23225, Pierce). After the samples were denatured using loading buffer, samples with equivalent protein concentrations were separated via SDS-PAGE and then transferred onto a PVDF membrane. The membranes were blocked with blocking buffer containing 5% skim milk and incubated with specific primary antibodies (as described above) at 4 °C overnight, followed by incubation with appropriate secondary HRP-conjugated antibodies. The bands were visualized using an ECL kit (#34077, Thermo Scientific).

### Statistical analysis

All data are presented as mean ± standard error of the mean (SEM). Comparisons between two groups and multiple groups were performed using the Student’s t-test and one-way analysis of variance, respectively. Statistical significance was set at *p* < 0.05. Statistical analyses were performed using the GraphPad Prism 6 software.

## Results

### Caspase-3 deficiency induces increased myelopoiesis and an aberrant hematopoietic stem cell pool in mice

**Fig. 1:**
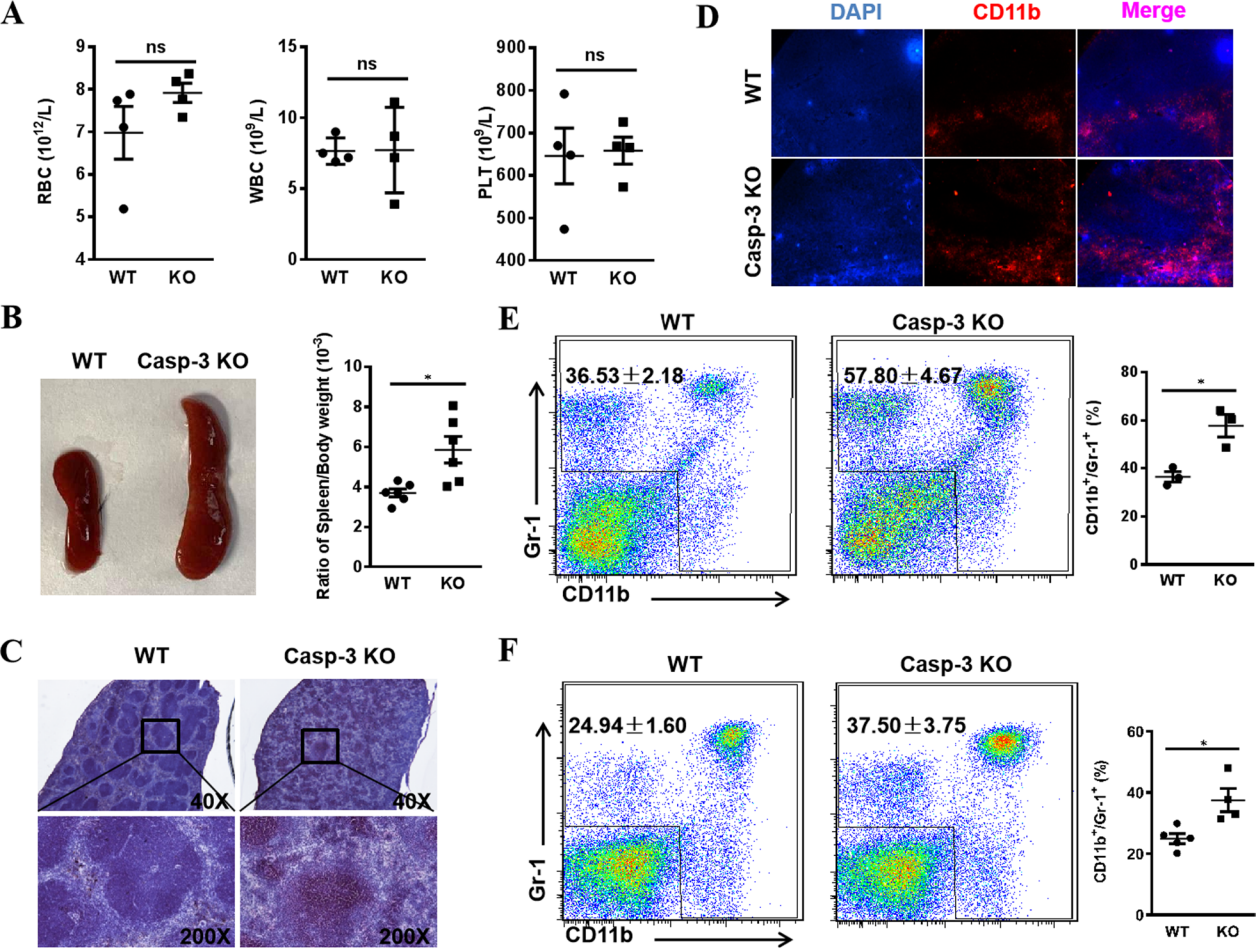
Myeloid lineage analysis in the peripheral blood and spleen of Casepase-3 gene knockout mice. **A** Analysis of peripheral blood counts of red blood cells (RBC), white blood cells (WBC), and platelets (PLT) in wild-type (WT) and Caspase-3^−/−^ (KO) mice (*n* = 4). **B** Spleen from WT and Caspase-3 KO mice and analysis of spleen index (the ratio of spleen/body weight) in WT and Caspase-3 KO mice (*n* = 6). **C** The H&E staining of spleen sections from WT and Caspase-3 KO mice. **D** The immunofluorescence staining of spleen sections from WT and Caspase-3 KO mice (DAPI: blue; CD11b: red). **E** Flow cytometry analysis of the percentage of CD11b^+^/Gr-1^+^ myeloid cells in the spleen of WT and Caspase-3 KO mice (*n* = 3). **F** Flow cytometry analysis of the percentage of CD11b^+^/Gr-1^+^ myeloid cells in the peripheral blood of WT and Caspase-3 KO mice (WT: *n* = 5; KO: *n* = 4). All data are presented as mean ± SEM. ns: not significant, *P < 0.05

To determine the in vivo regulatory function of Caspase-3, we generated Caspase-3^−/−^ mice and analyzed hematopoiesis in the peripheral circulation. Routine blood examination revealed that the number of red blood cells, white blood cells, and platelets did not change in the PB (Fig. 1A). We then analyzed hematopoiesis in the spleen, as it is an important peripheral immune and hematopoietic organ. As shown in Fig. 1B, Caspase-3^−/−^ mice exhibited an enlarged spleen and a significant increase in the spleen index. H&E staining showed pathological changes in the splenic architecture of Caspase-3^−/−^ mice, with a diffuse germinal center in the white pulp and a distorted boundary (Fig. 1C). Moreover, the intensity of the CD11b fluorescence signals was found to be higher in the spleen tissues of Caspase-3^−/−^ mice than in WT littermates (Fig. 1D). Flow cytometry analysis of spleen tissue samples from Caspase-3^−/−^ mice revealed a significant increase in the frequency of CD11b^+^/Gr-1^+^ myeloid cells and a decrease in the frequency of B lymphocytes (Fig. 1E, Additional file 2: Fig. S1A). These data suggest that increased myelopoiesis occurred in the spleen of Caspase-3^−/−^ mice. Similarly, an increase in the frequency of CD11b^+^/Gr-1^+^ myeloid cells was also noted in the PB of Caspase-3^−/−^ mice but the frequency of T and B lymphocytes appeared normal (Fig. 1F, Additional file 2: Fig. S1B), indicating that the absence of Caspase-3 led to increased myeloid cell output in the PB. Altogether, these results demonstrate that Caspase-3 deficiency induces myeloid lineage expansion in the PB and spleen.

**Fig. 2:**
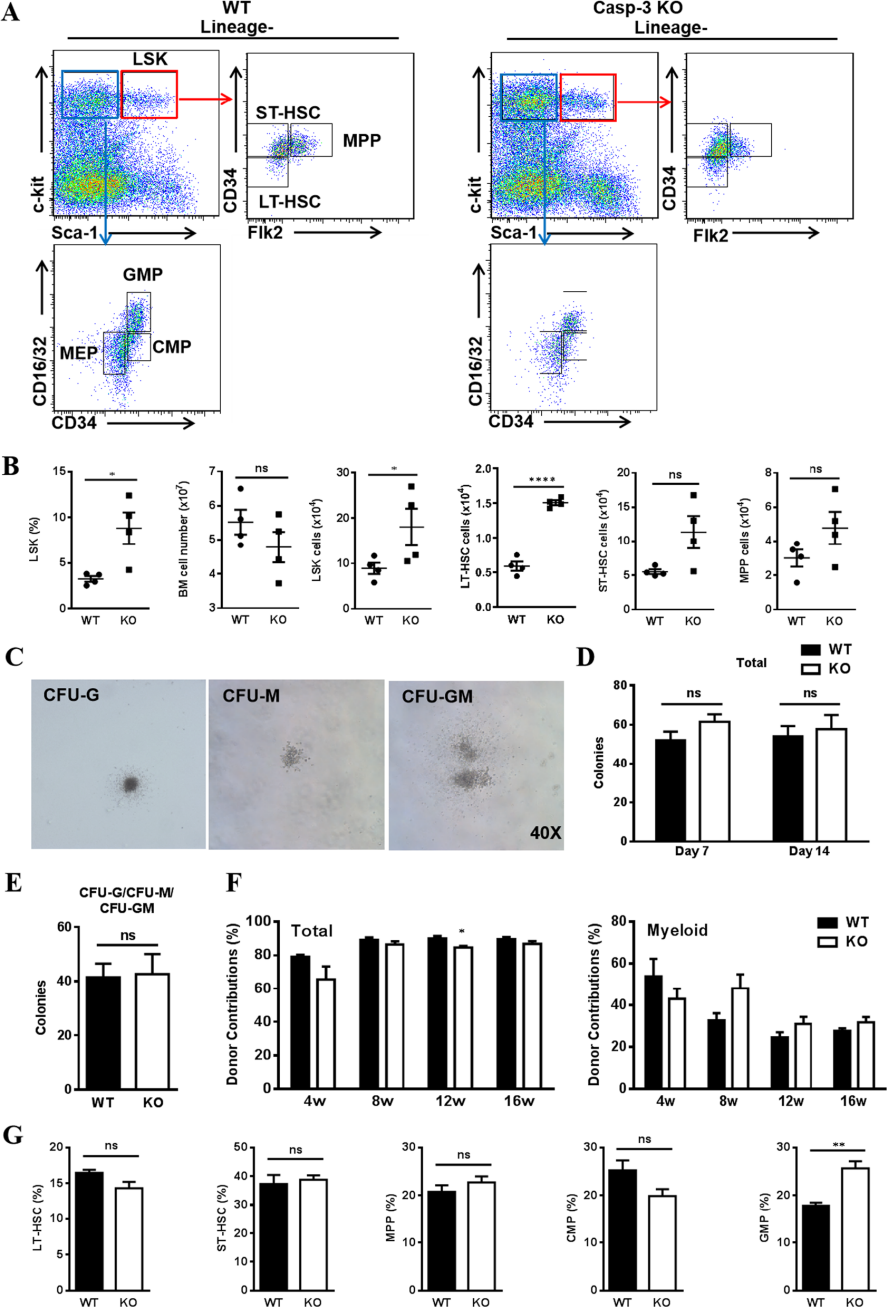
Hematopoietic progenitors detection in Casepase-3 gene knockout mice. **A** Flow cytometry plots of bone marrow cells from wild-type (WT) and Caspase-3^−/−^ (KO) mice, showing the gates on LSK (Lin^−^Sca-1^+^c-kit^+^), LT-HSC (Lin^−^Sca-1^+^c-kit^+^CD34^low^Flk2^low^), ST-HSC (Lin^−^Sca-1^+^c-kit^+^CD34^+^Flk2^low^), MPP (Lin^−^Sca-1^+^c-kit^+^CD34^+^Flk2^+^), CMP (Lin^−^Sca-1^−^c-kit^+^CD34^+^CD16/32^low^), GMP(Lin^−^Sca-1^−^c-kit^+^CD34^+^CD16/32^+^), and MEP(Lin^−^Sca-1^−^c-kit^+^CD34 ^low^ CD16/32^low^). **B** Flow cytometry analysis of the percentage of LSK cells and the number of total cells, LSK, LT-HSC, ST-HSC, and MPP in the bone marrow of WT and Caspase-3 KO mice (*n* = 4). **C** Morphology of colony-forming units of granulocyte–macrophage colonies (CFU-G, CFU-M, and CFU-GM). **D** Analysis of total colony numbers (including CFU-E, BFU-E, CFU-G, CFU-M, CFU-GM, and CFU-GEMM) at 7 and 14 days for the detection of HSPCs isolated from WT and Caspase-3 KO mice (*n* = 3–5). **E** Analysis of myeloid colony numbers (including CFU-G, CFU-M, and CFU-GM) at 14 days for the detection of HSPCs isolated from WT and Caspase-3 KO mice (WT: *n* = 4; KO: *n* = 3). (F-G) Bone marrow cells from WT and Caspase-3 KO mice (CD45.2) were isolated and injected into lethally irradiated recipients (CD45.1). Engraftment efficiency in recipient mice was assessed according to the donor contribution of CD45.2^+^ cells by flow cytometry. **F** The analysis of peripheral blood cells was performed every four weeks post-transplantation (WT: *n* = 3; KO: *n* = 5). **G** The assessment of progenitor subpopulations in the bone marrow was performed at 16 weeks after mice sacrifice (WT: *n* = 3; KO: *n* = 5). All data are presented as mean ± SEM. ns: not significant, *P < 0.05, **P < 0.01, ****P < 0.0001

Next, we investigated whether Caspase-3 regulates hematopoiesis in the BM. We found that the frequency and number of Lin^−^Sca-1^+^c-kit^+^ (LSK) cells were doubled in Caspase-3^−/−^ mice when compared to WT mice (Fig. 2A,B), which was consistent with the observations made by Scadden et al*.* [22]. In addition, the number of Lin^−^Sca-1^+^c-kit^+^CD34^low^Flk2^low^ long-term HSCs (LT-HSCs) was found to be significantly increased, while the primitive subpopulations of Lin^−^Sca-1^+^c-kit^+^CD34^+^Flk2^low^ short-term HSCs (ST-HSCs) and Lin^−^Sca-1^+^c-kit^+^CD34^+^Flk2^+^ multipotent progenitors (MPPs) were not significantly increased. These data indicate that the absence of Caspase-3 altered the composition of primitive hematopoietic populations. As results demonstrated that Caspase-3 deletion induced aberrant hematopoiesis in mice, we wondered if the function of HSPCs was also influenced. Thus, we evaluated the in vitro colony formation ability of Caspase-3^−/−^ HSPCs. By identifying and counting colony-forming units (CFUs), we found that the number of total colonies and myeloid colonies formed by Caspase-3^−/−^ HSPCs was comparable to that of the WT group (Fig. 2C,D,E). Moreover, the hematopoietic reconstitution ability of Caspase-3^−/−^ HSPCs was investigated by performing BM transplantation from Caspase-3^−/−^ mice into lethally irradiated WT recipients. We observed a slightly reduced percentage of donor-derived cells when analyzing the total cell population and T lymphocytes, but comparable myeloid lineage contributions (Fig. 2F, Additional file 2: Fig. S1C). Analysis of the BM composition at 16 weeks post-transplantation suggested that only the frequency of granulocyte–macrophage progenitors (GMPs) was significantly increased (Fig. 2G). Thus, these data imply that the function of Caspase-3 deficient HSPCs is not strikingly altered in regards to hematopoietic reconstitution.

### Caspase-3 deletion in BA-MSCs affects myeloid lineage development

**Fig. 3:**
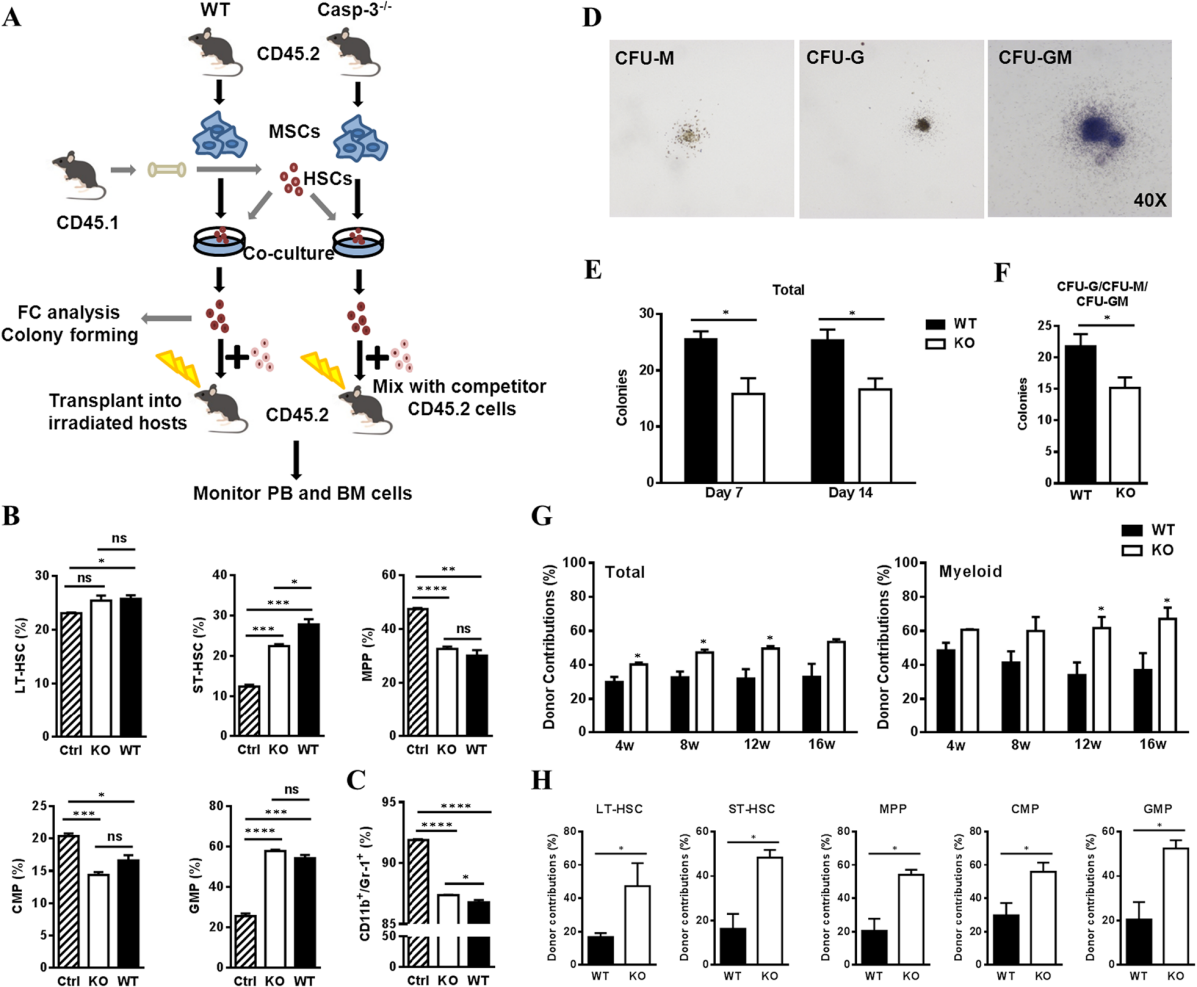
Hematopoiesis analysis after co-culture with Casepase-3^−/−^ BA-MSCs. **A** Schematic of the experimental procedures. The co-culture of HSPCs from wild-type (WT) mice (CD45.1) and BA-MSCs from Caspase-3^−/−^ (KO) mice (CD45.2) was performed in MEM-alpha medium. After 48 h, suspending HSPCs were harvested for flow cytometry and colony-forming assay. Ctrl: HSPCs cultured alone; KO: HSPCs cultured with Caspase-3^−/−^ BA-MSCs; WT: HSPCs cultured with WT BA-MSCs. In addition, the obtained HSPCs (CD45.1) were mixed with WT helper cells (CD45.2), and injected into 8.0 Gy-irradiated recipient mice (CD45.2) through ophthalmic vein. Engraftment efficiency in recipients was monitored by donor contribution of CD45.1^+^ cells in the peripheral blood and bone marrow. **B** Flow cytometry analysis of the percentage of LT-HSC, ST-HSC, MPP, CMP and GMP after co-culture (*n* = 3). **C** Flow cytometry analysis of the percentage of CD11b^+^/Gr-1^+^ myeloid cells after co-culture (*n* = 3). **D** Morphology of colony-forming units of granulocyte–macrophage colonies (CFU-G, CFU-M, and CFU-GM). **E** Analysis of total colony numbers (including CFU-E, BFU-E, CFU-G, CFU-M, CFU-GM, and CFU-GEMM) at 7 and 14 days for the detection of HSPCs after co-culture (*n* = 5). **F** Analysis of myeloid colony numbers (including CFU-G, CFU-M, and CFU-GM) at 14 days for the detection of HSPCs after co-culture (*n* = 5). **G** The analysis of peripheral blood cells was performed every four weeks post-transplantation (WT: *n* = 4; KO: *n* = 3). **H** The assessment of progenitor subpopulations in the bone marrow was performed at 16 weeks after mice sacrifice (WT: *n* = 4; KO: *n* = 3). All data are presented as mean ± SEM. ns: not significant, *P < 0.05, **P < 0.01, ***P < 0.001, ****P < 0.0001

As Caspase-3 deficiency induced increased myelopoiesis in mice but Caspase-3^−/−^ HSPCs did not produce more myeloid cells in irradiated hosts, we next investigated whether Caspase-3 deficient niche cells could regulate hematopoiesis. Previously, we identified BA-MSCs and proved that several BA-MSCs subpopulations could regulate hematogenesis [34]. Here, we isolated BA-MSCs from Caspase-3^−/−^ mice and co-cultured them with WT HSPCs. After 48 h, the suspended HSPCs from the upper layer of the co-culture plates were harvested and analyzed both in vivo and ex vivo (Fig. 3A). Flow cytometry analysis revealed that the frequency of primitive subpopulations in both the WT and KO groups was significantly different from that in the control group, indicating that the niche affected the composition of the hematopoietic stem cell pool (Fig. 3B). Notably, the phenotype of HSPCs co-cultured with Caspase-3^−/−^ BA-MSCs was more similar to that of the control group, which exhibited a default pathway of HSC differentiation in the absence of niche signals. Moreover, a slight increase in the CD11b^+^/Gr-1^+^ myeloid population was observed in the KO group when compared to that in the WT group (Fig. 3C). These data suggest that Caspase-3 deficient BA-MSCs may regulate myelopoiesis. The in vitro colony formation assay revealed that HSPCs co-cultured with Caspase-3^−/−^ BA-MSCs generated a fewer total number of colonies, especially CFU-G/CFU-M/CFU-GM (Fig. 3D,E,F).

In order to determine whether Caspase-3^−/−^ BA-MSCs had an impact on the hematopoietic reconstitution ability of HSPCs, we performed a competitive BM transplantation assay (Fig. 3A). The survival rate of mice in the KO group post-transplantation was shown to be reduced when compared to the WT group (Figure Additional file 2: Fig. S2A), indicating that Caspase-3^−/−^ BA-MSCs impaired the long-term reconstituting capability of HSPCs. We also found that Caspase-3 deficiency in BA-MSCs led to an increased donor contribution of total cells in the PB of recipients, which was also accompanied by myeloid-biased differentiation (Fig. 3G, Additional file 2: Fig. S2B). Meanwhile, an increased frequency of LSK and primitive subpopulations was observed in the BM (Fig. 3H). Collectively, these data demonstrate that deletion of Caspase-3 in BA-MSCs regulates the balance of the hematopoietic stem cell pool and induces a biased myeloid commitment of HSPCs.

### Caspase-3 deletion did not affect apoptosis but reduced SCF and CXCL12 levels in BA-MSCs

**Fig. 4:**
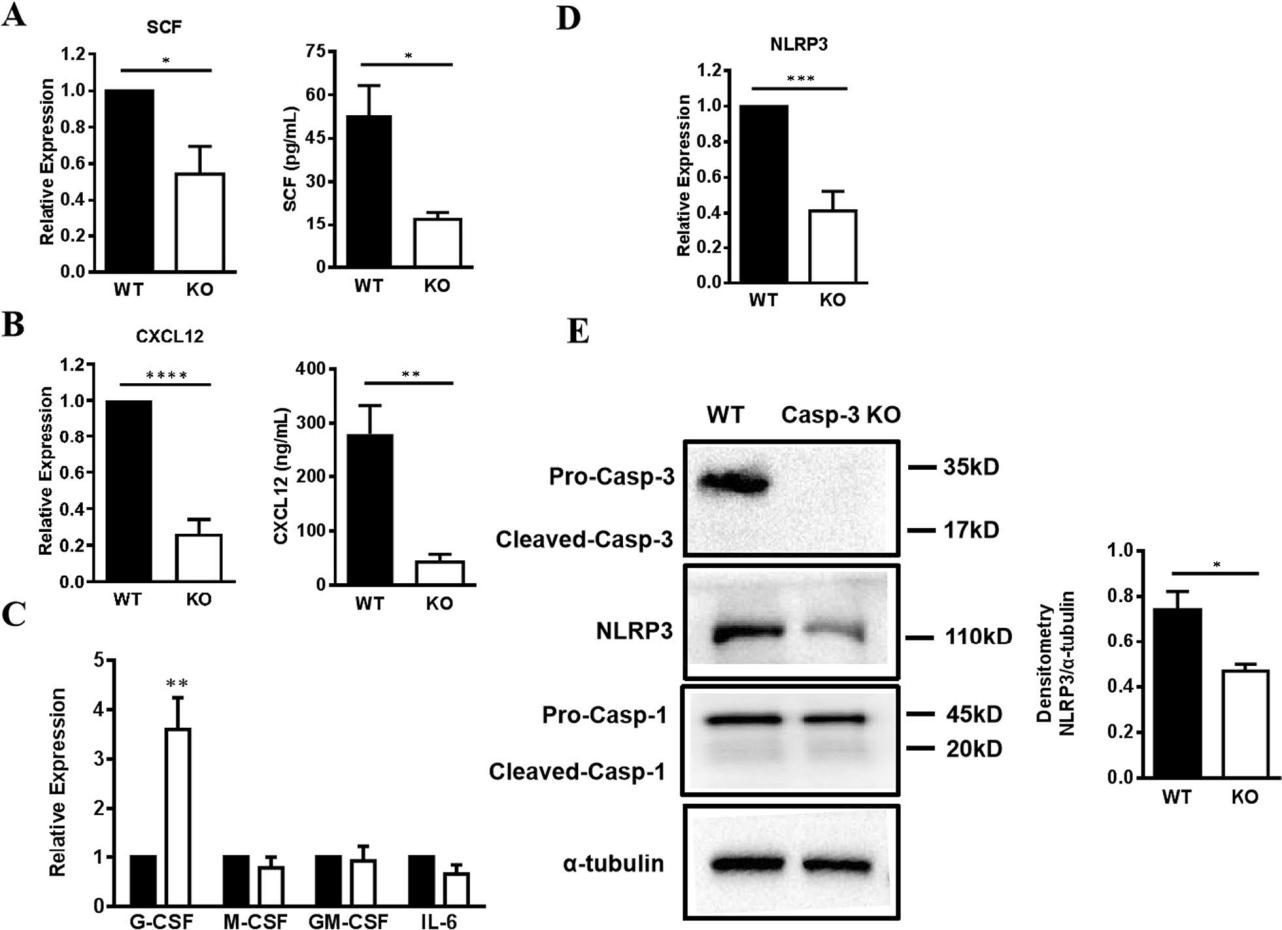
SCF and CXCL12 level determination in BA-MSCs after Casepase-3 gene knockout. **A** Gene expression of SCF in wild-type (WT) and Caspase-3^−/−^ (KO) BA-MSCs by qRT-PCR (*n* = 6), and ELISA analysis in the supernatants after culture for 7 days (*n* = 4). **B** Gene expression of CXCL12 in WT and Caspase-3^−/−^ BA-MSCs by qRT-PCR (*n* = 6), and ELISA analysis in the supernatants after culture for 7 days (*n* = 4). **C** Gene expression of G-CSF, M-CSF, GM-CSF, and IL-6 in WT and Caspase-3^−/−^ BA-MSCs by qRT-PCR (*n* = 3–4). **D** Gene expression of NLRP3 in WT and Caspase-3^−/−^ BA-MSCs by qRT-PCR (*n* = 5). **E** Western blot analysis of the expression of Caspase-3, NLRP3, and Caspase-1 in WT and Caspase-3^−/−^ BA-MSCs (*n* = 3). All data are presented as mean ± SEM. ns: not significant, *P < 0.05, **P < 0.01, ***P < 0.001, ****P < 0.0001

Considering that Caspase-3 deficiency in BA-MSCs modulated hematopoiesis and promoted myeloid lineage expansion, we aimed to investigate the underlying mechanisms by analyzing cytokine secretion in BA-MSCs. SCF and SDF-1/CXCL12 are important cytokines that help maintain HSCs and hematopoietic progenitors in the niche [14]. Notably, qRT-PCR analysis revealed that SCF and CXCL12 mRNA levels were markedly decreased in Caspase-3^−/−^ BA-MSCs (Fig. 4A,B). The downregulation of these two retention factors was further verified by ELISA (Fig. 4A,B), suggesting that Caspase-3^−/−^ BA-MSCs exhibit a reduced capacity of maintaining HSCs and hematopoietic progenitors.

Next, we determined the cell cycle stages of HSPCs after co-culture with Caspase-3^−/−^ BA-MSCs. The data revealed a shift from G0/G1 towards S + G2/M, suggesting that co-culture with Caspase-3^−/−^ BA-MSCs induced a higher percentage of cells to enter the mitotic stage of the cell cycle (Additional file 2: Fig. S3A). Annexin V/PI apoptosis analysis showed a decreased percentage of Annexin V^−^PI^−^ viable cells but an increased ratio of Annexin V^+^ apoptotic cells (Additional file 2: Fig. S3B). These results suggest that Caspase-3 deficiency in BA-MSCs strikingly reduced quiescence and induced apoptosis of HSPCs, which may be due to the reduced levels of SCF and CXCL12. In contrast, no significant difference was observed between WT and Caspase-3^−/−^ BA-MSCs, indicating that Caspase-3 did not alter the cell cycle and apoptosis of BA-MSCs (Additional file 2: Fig. S3C,D,E). Additionally, we analyzed the gene expression levels of several myeloid cytokines and found a robust increase in the expression of G-CSF (Fig. 4C), which may promote myeloid lineage development. Collectively, these results suggest that Caspase-3 deletion does not affect apoptosis and cell cycle in BA-MSCs but reduces the expression of hematopoietic cytokines, including SCF and CXCL12.

To further verify our hypothesis, we supplemented recombinant SCF and CXCL12 in the Caspase-3^−/−^ co-culture system to rescue the phenotype of Caspase-3 deficiency in vitro. Flow cytometry analysis revealed that the frequency of LSK and Lin^−^Sca-1^−^c-kit^+^ cells was significantly increased when SCF and CXCL12 additionally added in the Caspase-3^−/−^ BA-MSCs co-culture medium, compared to the WT group (Additional file 2: Fig. S4A). Specifically, the frequency of LT-HSCs and ST-HSCs was slightly increased, while the frequency of MPPs was reduced. More CMPs and less GMPs were also observed after SCF and CXCL12 supplemented. Moreover, a decrease in the frequency of CD11b^+^/Gr-1^+^ myeloid population was noted (Additional file 2: Fig. S4B). These data indicate that the supplemented SCF and CXCL12 in Caspase-3^−/−^ BA-MSCs co-culture system support the retention of HSCs. Overall, these findings suggest that recombinant SCF and CXCL12 rescue the phenotype of Caspase-3 deficiency in vitro.

### Phenotypes of NLRP3 deficient mice were similar to those of Caspase-3 gene knockout mice

**Fig. 5:**
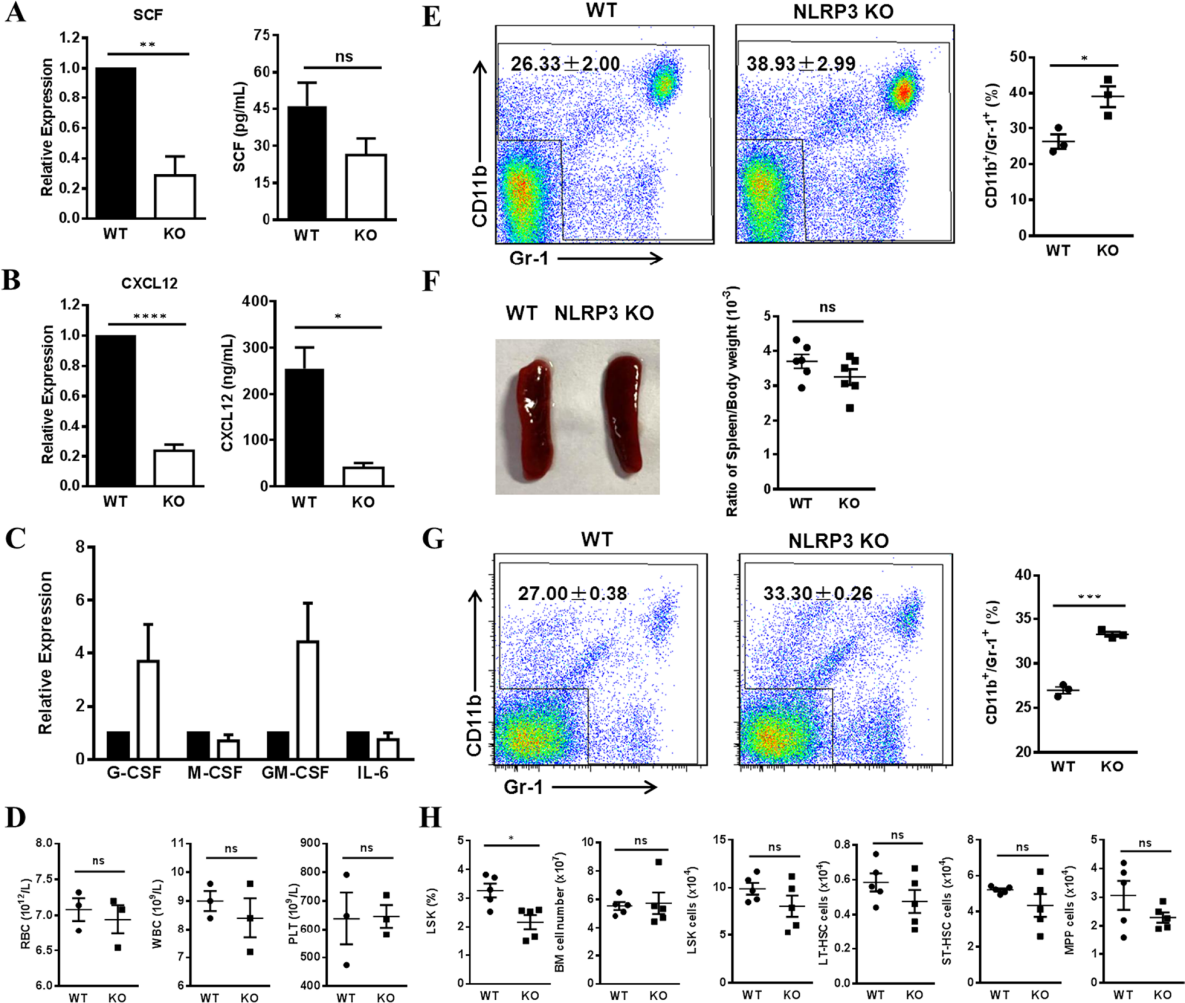
Myeloid lineage analysis in mice after NLRP3 gene knockout. **A** Gene expression of SCF in wild-type (WT) and NLRP3^−/−^ (KO) BA-MSCs by qRT-PCR (*n* = 4), and ELISA analysis in the supernatants after culture for 7 days (*n* = 3). **B** Gene expression of CXCL12 in WT and NLRP3^−/−^ BA-MSCs by qRT-PCR (*n* = 4), and ELISA analysis in the supernatants after culture for 7 days (*n* = 3). **C** Gene expression of G-CSF, M-CSF, GM-CSF, and IL-6 in WT and NLRP3^−/−^ BA-MSCs by qRT-PCR (*n* = 4). **D** Analysis of peripheral blood counts of red blood cells (RBC), white blood cells (WBC) and platelets (PLT) in WT and NLRP3 KO mice (*n* = 3). **E** Flow cytometry analysis of the percentage of CD11b^+^/Gr-1^+^ myeloid cells in the peripheral blood of WT and NLRP3 KO mice (*n* = 3). **F** Spleen from WT and NLRP3 KO mice and analysis of spleen index (the ratio of spleen/body weight) in WT and NLRP3 KO mice (*n* = 6). **G** Flow cytometry analysis of the percentage of CD11b^+^/Gr-1^+^ myeloid cells in the spleen of WT and NLRP3 KO mice (*n* = 3). **H** Flow cytometry analysis of the percentage of LSK cells and the number of total cells, LSK, LT-HSC, ST-HSC, and MPP in the bone marrow of WT and NLRP3 KO mice (*n* = 5). All data are presented as mean ± SEM. ns: not significant, *P < 0.05, **P < 0.01, ***P < 0.001, ****P < 0.0001

It has been observed in clinical practice that inflammation regulates hematopoiesis [35–37]. As an important player in the inflammatory response, NLRP3 is thought to be involved in myeloid development [29]. Thus, we investigated whether NLRP3 participates in hematopoiesis through BA-MSCs. We first evaluated the expression of NLRP3 in Caspase-3^−/−^ BA-MSCs using qRT-PCR and western blot and found that NLRP3 was markedly downregulated (Fig. 4D,E), suggesting that Caspase-3 deletion reduced NLRP3 levels in BA-MSCs. We then isolated BA-MSCs from NLRP3 deficient mice and analyzed the expression of cytokines associated with hematopoiesis. Interestingly, SCF and CXCL12 levels were found to be decreased in NLRP3^−/−^ BA-MSCs (Fig. 5A,B), which is similar to the results obtained in Caspase-3^−/−^ BA-MSCs. Moreover, the gene expression levels of myeloid cytokines (including G-CSF and GM-CSF) were found to be not-significantly upregulated (Fig. 5C), which might be supportive of myeloid expansion. Therefore, we hypothesized that the expression of NLRP3 in BA-MSCs may play a role in hematopoiesis.

To determine how NLRP3 regulates hematopoiesis alongside Caspase-3, we first analyzed hematopoiesis in NLRP3^−/−^ mice. No significant difference was observed in the number of red blood cells, white blood cells, and platelets in the PB between WT and NLRP3^−/−^ mice (Fig. 5D). Surprisingly, NLRP3^−/−^ mice exhibited a significant increase in the percentage of CD11b^+^/Gr-1^+^ myeloid cells in the PB but had normal percentages of T and B lymphocytes (Fig. 5E, Additional file 2: Fig. S5A), which was similar to the phenotype observed in Caspase-3^−/−^ mice. Although splenic enlargement was not observed in NLRP3^−/−^ mice (Fig. 5F), we observed a marked increase in the frequency of myeloid cells in the spleen (Fig. 5G, Additional file 2: Fig. S5B).Thus, these data suggest that the absence of NLRP3 induced myeloid expansion in the PB and spleen. We further examined hematopoiesis in the BM of NLRP3^−/−^ mice and found that the frequency of LSK cells was significantly reduced (Fig. 5H). However, we did not find any differences in the number of HSCs and MPPs when compared with WT mice (Fig. 5H). Taken together, these findings suggest that NLRP3 deficiency leads to increased myelopoiesis, which is similar to the phenotypes observed in Caspase-3^−/−^ mice.

### NLRP3 deficient BA-MSCs regulate hematopoiesis by affecting the cell cycle and apoptosis of HSPCs

**Fig. 6:**
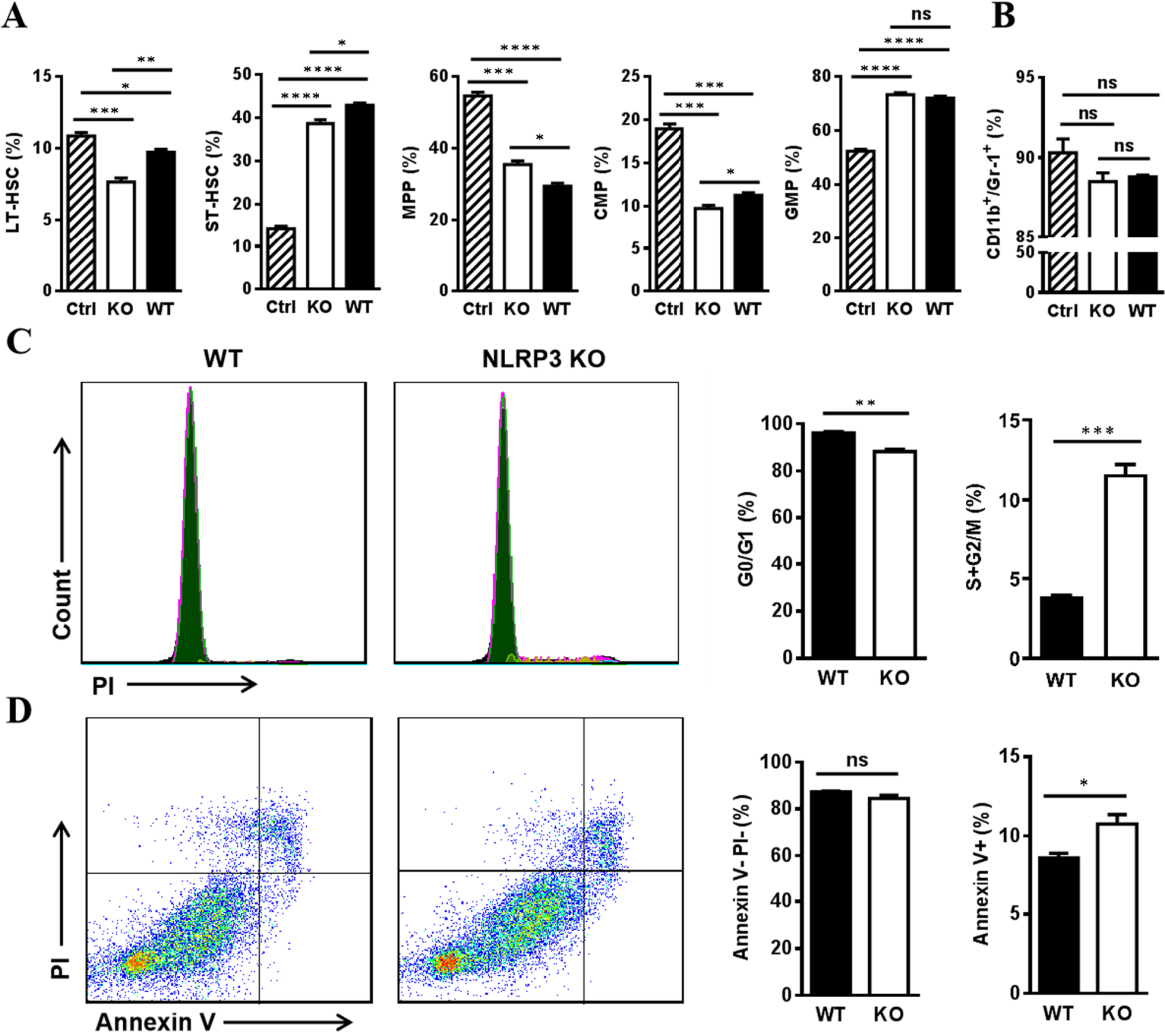
Cell cycle and apoptosis analysis of HSPCs after co-culture with NLRP3^−/−^ BA-MSCs. (A-B) The co-culture of HSPCs from wild-type (WT) mice and BA-MSCs from NLRP3^−/−^ (KO) mice was performed in MEM-alpha medium. After 48 h, suspending HSPCs were harvested for analysis. Ctrl: HSPCs cultured alone; KO: HSPCs cultured with NLRP3^−/−^ BA-MSCs; WT: HSPCs cultured with WT BA-MSCs. **A** Flow cytometry analysis of the percentage of LT-HSC, ST-HSC, MPP, CMP, and GMP after co-culture (*n* = 3). **B** Flow cytometry analysis of the percentage of CD11b^+^/Gr-1^+^ myeloid cells after co-culture (*n* = 3). **C** Cell cycle analysis of HSPCs after co-culture with WT and NLRP3^−/−^ BA-MSCs (*n* = 3). **D** Apoptosis analysis of HSPCs after co-culture with WT and NLRP3^−/−^ BA-MSCs (*n* = 3). All data are presented as mean ± SEM. ns: not significant, *P < 0.05, **P < 0.01, ***P < 0.001, ****P < 0.0001

As the expression of cytokines associated with hematopoiesis was found to be altered in BA-MSCs after NLRP3 deletion, we further investigated the function of NLRP3 deficient BA-MSCs in hematopoiesis regulation by co-culturing NLRP3^−/−^ BA-MSCs and WT HSPCs. Flow cytometry analysis revealed that after co-culture with NLRP3^−/−^ BA-MSCs, the frequency of primitive subpopulations (including LT-HSCs, ST-HSCs, MPPs, and CMPs) was significantly different from that observed in the WT group (Fig. 6A). Moreover, the composition of HSPCs co-cultured with NLRP3^−/−^ BA-MSCs was similar to that of the control group. Therefore, the data indicate that deletion of NLRP3 in BA-MSCs impaired steady-state hematopoiesis. However, no significant difference was observed in the frequency of myeloid populations (Fig. 6B). Next, we investigated the cell cycle and apoptosis of HSPCs after co-culture with NLRP3^−/−^ BA-MSCs. We found that more cells entered the mitotic stage (S + G2/M) of the cell cycle (Fig. 6C) and we also found a higher percentage of Annexin V^+^ apoptotic cells (Fig. 6D). Thus, these results suggest that NLRP3 deficiency in BA-MSCs impairs quiescence and induces apoptosis of HSPCs.

Previously, we identified a mesenchymal stromal progenitor hierarchy of CD45^−^Ter119^−^CD31^−^CD166^−^CD146^−^Sca1^+^ (Sca1^+^) cells and showed that Sca1^+^ progenitors could generate CD146^+^ intermediate and CD166^+^ mature osteoprogenitors [34]. Thus, we next explored the effect of NLRP3 on MSC subpopulations. Sca1^+^ BA-MSCs were isolated from WT mice, sorted using magnetic beads, and cultured in MEM-alpha medium. During the cell culture period in which Sca1^+^ progenitors gradually produced CD146^+^ and CD166^+^ cells, we found that the gene expression of NLRP3 was significantly upregulated (Additional file 2: Fig. S6A). Simultaneously, the secretion of SCF and CXCL12 was also shown to be increased (Additional file 2: Fig. S6B), implying that NLRP3 might regulate the hematopoietic cytokine levels of MSC subpopulations. Furthermore, we evaluated the frequency of three MSC subpopulations in NLRP3 deficient mice and noticed that the percentage of CD166^+^ cells was markedly reduced, while the percentages of Sca1^+^ and CD146^+^ cells remained unchanged (Additional file 2: Fig. S6C). This phenotype was consistent with our previous finding that SCF/CXCL12 levels are reduced in NLRP3^−/−^ BA-MSCs. Overall, these findings suggest that NLRP3 may regulate the levels of cytokines involved in hematopoiesis.

### Caspase-3 might regulate SCF and CXCL12 levels via NLRP3 in BA-MSCs

**Fig. 7:**
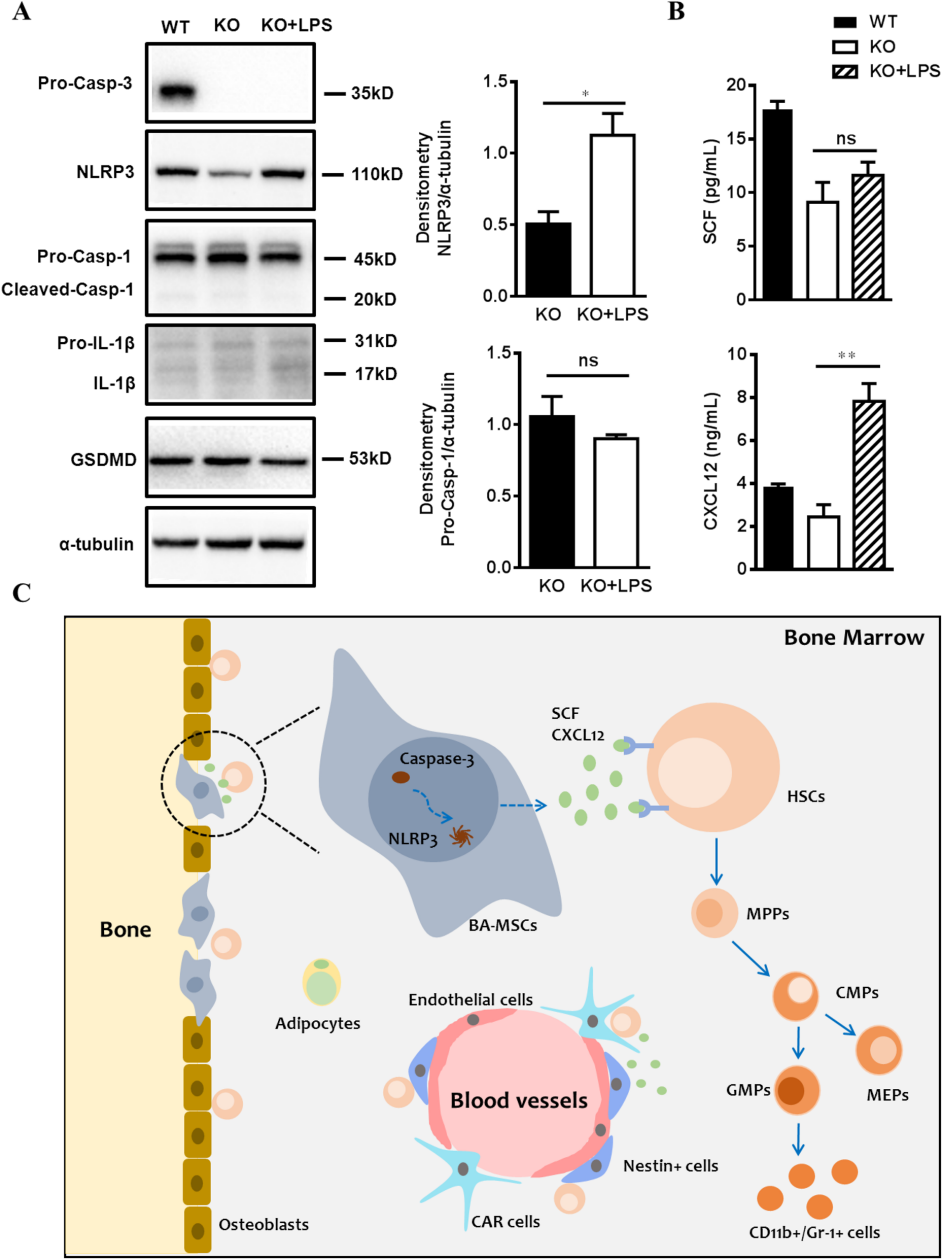
SCF and CXCL12 level determination in Casepase-3^−/−^ BA-MSCs after stimulated with LPS. **A** Western blot analysis of the expression of Caspase-3, NLRP3, Caspase-1, IL-1β, and GSDMD in WT BA-MSCs (WT), Caspase-3^−/−^ BA-MSCs (KO) and Caspase-3^−/−^ BA-MSCs stimulated with LPS for 72 h (KO + LPS) (*n* = 3). **B** ELISA analysis in the supernatants of WT BA-MSCs (WT), Caspase-3^−/−^ BA-MSCs (KO) and Caspase-3^−/−^ BA-MSCs stimulated with LPS for 72 h (KO + LPS) (*n* = 3–4). **C** The schematic summaries our findings that Caspase-3 might regulate SCF and CXCL12 levels via NLRP3 in BA-MSCs. All data are presented as mean ± SEM. ns: not significant, *P < 0.05, **P < 0.01

Given that both Caspase-3 and NLRP3 deficient BA-MSCs exhibited decreased SCF and CXCL12 levels and Caspase-3 deletion reduced NLRP3 expression in BA-MSCs, we sought to determine the interaction between Caspase-3 and NLRP3 in BA-MSCs and how that interaction regulates cytokine expression. We isolated BA-MSCs from Caspase-3^−/−^ mice and cultured them in vitro, after which lipopolysaccharide (LPS) was added to the culture medium to upregulate NLPR3 expression. Western blot analysis revealed that NLRP3 levels were significantly increased in Caspase-3^−/−^ BA-MSCs after stimulation with LPS for 72 h, while the expression of other inflammasome-related proteins (including Caspase-1, IL-1β, and GSDMD) remained unchanged (Fig. 7A). These results indicate that the NLRP3 inflammasome was not activated and pyroptosis was not induced under these conditions, while only NLRP3 expression was upregulated in Caspase-3^−/−^ BA-MSCs. Next, we evaluated SCF and CXCL12 levels by ELISA and found that they were increased in Caspase-3^−/−^ BA-MSCs after NLRP3 upregulation (Fig. 7B). As such, our results suggest that Caspase-3 might regulate SCF and CXCL12 levels via NLRP3 in BA-MSCs.

## Discussion

In the current study, we found that Caspase-3 deficiency induced increased myelopoiesis and an aberrant HSC pool in mice. Deletion of Caspase-3 in BA-MSCs regulated myeloid lineage development by reducing the secretion of hematopoietic cytokines, including SCF and CXCL12. NLRP3 was also shown to be potentially involved in the myeloid commitment by modulating the cell cycle and apoptosis of hematopoietic progenitors. Thus, the data presented here reveal that the Caspase-3/NLRP3 signaling is an important regulator of physiological hematopoiesis (Fig. 7C). Moreover, our findings are helpful in extending our knowledge of the involvement of niche signals in hematopoiesis regulation in the BM.

Caspase-3 is not only a critical enzyme in apoptosis but also an important mediator of cell proliferation, self-renewal, and differentiation. Previous studies have reported that Caspase-3 gene knockout mice had smaller body sizes than their WT counterparts [22]. The heterozygous Caspase-3 deficient mice exhibited symptoms of osteoporosis, which reduced bone mineral density (BMD) with age [38]. These findings suggest that Caspase-3 is involved in osteogenesis, which might be attributed to a significant defect in osteogenic differentiation of BM-MSCs. Notably, Liu et al*.* showed that a reduction in apoptotic body formation impaired both the osteogenic and adipogenic differentiation of BM-MSCs [39]. Furthermore, Caspase-3 silencing promoted cell proliferation and enhanced the anti-apoptotic capacity of BM-MSCs in hypoxic conditions in vitro [40]. These studies show that Caspase-3 plays a role in regulating the differentiation and proliferation of MSCs. In this study, we revealed that Caspase-3 also participates in mediating the expression of cytokines in MSCs. We found that the SCF and CXCL12 secretion of Caspase-3 deficient MSCs was significantly impaired, which induced aberrant composition of the HSC pool and myeloid development. However, the mechanism by which Caspase-3 affects the MSC cytokine secretion requires further investigation.

Among the hematopoietic cytokines produced by niche cells, SCF and CXCL12 contribute greatly to the maintenance of HSPCs in the BM. CAR cells have been proven to be major producers of these two retention factors [14]. HSCs from CAR cell-depleted mice were shown to exhibit biases in myeloid lineage differentiation [16], indicating that a lack of SCF and CXCL12 induces HSCs to increase myeloid output. Our results identified BA-MSCs as a part of the hematopoietic niche, which also produce SCF and CXCL12. Caspase-3 deficiency in BA-MSCs reduced the secretion of SCF and CXCL12 and led to myeloid expansion of HSCs, which is consistent with the phenotype of HSCs after CAR cell depletion [16]. Therefore, we hypothesize that BA-MSCs share some functional similarities with CAR cells, suggesting that BA-MSCs are important niche cells in the BM. Moreover, it has been observed that the myeloid-biased potential of HSCs is an intrinsic behavior in the absence of niche signals [2], which explains why a decrease in SCF and CXCL12 levels in the niche leads to increased myelopoiesis. Alternatively, BA-MSCs provide signals for the maintenance of HSCs.

The NLRP3 inflammasome is an important intracellular protein complex that drives immune responses and induces pyroptosis by releasing pro-inflammatory cytokines [24, 41]. Studies have demonstrated that the NLRP3 inflammasome is involved in the incidence of blood disorders, implying that it plays a role in hematopoiesis regulation. S100A9 mediates NLRP3 inflammasome activation in the HSPCs of patients with myelodysplastic syndrome (MDS), which induces clonal expansion and pyroptotic cell death [42, 43]. Salaro et al*.* investigated the P2X7/NLRP3 axis in lymphocytes from patients with chronic lymphocytic leukemia (CLL) and found that NLRP3 was significantly downregulated in CLL lymphocytes, indicating that NLRP3 is a negative regulator of leukemic cell proliferation [28]. Moreover, studies have reported that NLRP3 inflammasome activation may be associated with a poor prognosis in acute lymphoblastic leukemia (ALL) patients [44]. These studies provide evidence that the NLRP3 inflammasome participates in aberrant hematopoiesis under inflammatory conditions. In this study, we explored the role of NLRP3 in hematopoiesis regulation under physiological conditions, which has rarely been investigated. We generated NLRP3 gene knockout mice and analyzed hematopoiesis in the peripheral circulation and BM. We also explored the effect of NLRP3 deficient BA-MSCs on hematopoiesis regulation. Based on our observations, we inferred that NLRP3 plays a key role in physiological myeloid development. Thus, these findings extend our understanding of the regulatory function of NLRP3 in hematopoiesis under physiological conditions.

As NLRP3 gene knockout mice share some phenotypic similarities with Caspase-3-depleted mice and NLRP3 deficient BA-MSCs exhibited decreased levels of retention factors, similar to those observed in Caspase-3 deficient BA-MSCs, we hypothesized Caspase-3 and NLRP3 might interact in BA-MSCs in order to mediate cytokine secretion. By upregulating NLPR3 expression in Caspase-3-depleted BA-MSCs, we observed increased levels of SCF and CXCL12, which confirmed our preliminary speculation. However, the detailed molecular mechanisms require further clarification, which is one of the limitations of this study. Previous studies have demonstrated that Caspase-3 could induce the activation of the NLRP3 inflammasome via potassium efflux [26]. Moreover, when the canonical NLRP3 pathway is blocked, Caspase-3 can induce pyroptosis through the cleavage of GSDME [45, 46]. These studies suggest that there may be direct or indirect interactions between these two molecules. In future studies, we will explore the mechanisms underlying Caspase-3/NLRP3 signaling-mediated regulation of hematopoietic cytokine secretion in BA-MSCs.

It is important to perform the bone marrow transplantation from the wild-type mice into the lethally irradiated Caspase-3 or NLRP3 deficient mice to explore the function of microenvironment. Previously, we have performed the transplantation using Caspase-3^−/−^ mice as recipients, but the mice never survived more than a month after transplantation (data not shown). Then we switched to use heterozygous caspase-3 deficient (Caspase-3^±^) mice as recipients. Analysis of peripheral blood of recipients revealed a slight increase in engraftments when analyzing the total cell population and myeloid lineage contributions at 16 weeks post-transplantation (data not shown). These data implied that the Caspase-3 deficient niche could have some effects on hematopoiesis, but the function of caspase-3 in niche on hematopoiesis regulation is not fully explored. Our study here aimed to reveal the function of Caspase-3 and NLRP3 deficient MSCs in hematopoiesis regulation. To exclude the possibility that other niche signals mediate hematopoiesis through Caspase-3 or NLRP3, the most definitive experiment would be to generate MSC-specific gene knockout mice and perform the transplantation. Thus, the other limitation of the current study is that the conditional Caspase-3 and NLRP3 gene knockout mice were not used in the experiments, as we have not yet successfully established a MSC-specific gene mutation mouse model. Therefore, we isolated BA-MSCs from gene knockout animals and analyzed their function through co-culture with HSPCs in vitro. However, the hematopoietic phenotypes of Caspase-3 and NLRP3 gene knockout mice cannot be fully attributed to the function of BA-MSCs, as the effects of other niche cells were not investigated in this study. Therefore, we do not exclude the possibility that other niche signals mediate hematopoiesis through Caspase-3 or NLRP3.

## Conclusions

In summary, our findings reveal a novel function of the Caspase-3/NLRP3 signaling as a key regulator in physiological hematopoiesis, thereby extending our understanding of how microenvironmental niche signals regulate HSC maintenance and myeloid lineage development within the BM.

## Supplementary Information

**Additional file 1:** Supplementary tables.

**Additional file 2:** Supplementary figures.

## Data Availability

The datasets used and/or analyzed during the current study are available from the corresponding author on reasonable request.

## Abbreviations

ACK: Ammonium-Chloride-Potassium
ALL: Acute lymphoblastic leukemia
ASCs: Adipose-derived stromal cells
BA-MSCs: Bone-associated mesenchymal stromal cells
BM: Bone marrow
BMD: Bone mineral density
CAR cells: CXCL12-abundant reticular cells
CFUs: Colony-forming units
CLL: Chronic lymphocytic leukemia
CMPs: Common myeloid progenitors
DAMPs: Damage-associated molecular patterns
FBS: Fetal bovine serum
GMPs: Granulocyte–macrophage progenitors
HSCs: Hematopoietic stem cells
HSPCs: Hematopoietic stem/progenitor cells
H&E staining: Hematoxylin–eosin staining
LPS: Lipopolysaccharide
LT-HSCs: Long-term hematopoietic stem cells
MPPs: Multipotent progenitors
MSCs: Mesenchymal stromal cells
PAMPs: Pathogen-associated molecular patterns
PB: Peripheral blood
PBS: Phosphate-buffered saline
PFA: Paraformaldehyde
SCF: Stem cell factor
SDF-1/CXCL12: Stromal cell-derived factor 1
ST-HSCs: Short-term hematopoietic stem cells
WT: Wild-type
