# Supplementary material for: Caspase-3/NLRP3 signaling in the mesenchymal stromal niche regulates myeloid-biased hematopoiesis
Source: Stem Cell Res Ther. 2021 Nov 20;12:579. doi: 10.1186/s13287-021-02640-y (PMC8605603; doi:10.1186/s13287-021-02640-y)
Supplement: Supplementary file 1 — Additional file 1: Supplementary tables. [file 13287_2021_2640_MOESM1_ESM.docx]

**Figure S1. Lymphoid lineage analysis in the peripheral blood, spleen and bone marrow of Casepase-3 gene knockout mice.**

(A) Flow cytometry analysis of the percentage of T and B lymphocytes in the spleen of WT and Caspase-3 KO mice (n=5). (B) Flow cytometry analysis of the percentage of T and B lymphocytes in the peripheral blood of wild-type (WT) and Caspase-3^-/-^ (KO) mice (WT: n=5; KO: n=4). (C) Bone marrow cells from WT and Caspase-3 KO mice (CD45.2) were isolated and injected into lethally irradiated recipients (CD45.1) through ophthalmic vein. Engraftment efficiency in recipient mice was assessed by donor contribution of CD45.2^+^ cells by flow cytometry. The analysis of peripheral blood lymphocytes was performed every four weeks post-transplantation (WT: n=3; KO: n=5). All data are presented as mean±SEM. ns: not significant, **P*<0.05, ***P*<0.01.

**Figure S2.** **Survival curve and lymphoid phenotypes of recipients after transplantation.**

(A-B) The co-culture of HSPCs from wild-type (WT) mice (CD45.1) and BA-MSCs from Caspase-3^-/-^ (KO) mice (CD45.2) was performed in MEM-alpha medium. The harvested HSPCs (CD45.1) were mixed with WT helper cells (CD45.2), and transfused into 8.0 Gy-irradiated recipient mice (CD45.2). Engraftment efficiency in recipients was monitored by donor contribution of CD45.1^+^ cells in peripheral blood and bone marrow. (A) The survival of recipient mice was analyzed after transplantation. (B) The analysis of peripheral blood lymphocytes was performed every four weeks post-transplantation (WT: n=4; KO: n=3). All data are presented as mean±SEM. ns: not significant, ***P*<0.01.

**Figure S3. Cell cycle and apoptosis analysis of HSPCs after co-culture with Caspase-3^-/-^ BA-MSCs.**

(A) Cell cycle analysis of HSPCs after co-culture with wild-type (WT) and Caspase-3^-/-^ (KO) BA-MSCs (n=3). (B) Apoptosis analysis of HSPCs after co-culture with WT and Caspase-3^-/-^ BA-MSCs (n=3). (C) Cell cycle analysis of WT and Caspase-3^-/-^ BA-MSCs (n=3). (D) Apoptosis analysis of WT and Caspase-3^-/-^ BA-MSCs (n=3). (E) Western blot analysis of the expression of Caspase-3 and PARP in WT and Caspase-3^-/-^ BA-MSCs (n=3). All data are presented as mean±SEM. ns: not significant, **P*<0.05.

**Figure S4. Hematopoiesis analysis of HSPCs after rescue with recombinant SCF and CXCL12**

(A-B) The co-culture of HSPCs from wild-type (WT) mice and BA-MSCs from Caspase-3^-/-^ (KO) mice was performed in MEM-alpha medium supplemented with recombinant SCF and CXCL12. After 48 hours, suspending HSPCs were harvested for analysis. WT+SCF/CXCL12: HSPCs cultured with WT BA-MSCs in MEM-alpha medium supplemented with recombinant SCF and CXCL12; WT: HSPCs cultured with WT BA-MSCs; KO+SCF/CXCL12: HSPCs cultured with Caspase-3^-/-^ BA-MSCs in MEM-alpha medium supplemented with recombinant SCF and CXCL12; KO: HSPCs cultured with Caspase-3^-/-^ BA-MSCs. (A) Flow cytometry analysis of the percentage of LSK, Lin^-^Sca-1^-^c-kit^+^, LT-HSC, ST-HSC, MPP, CMP, and GMP after co-culture (n=3). (B) Flow cytometry analysis of the percentage of CD11b^+^/Gr-1^+^ myeloid cells after co-culture (n=3). All data are presented as mean±SEM. ns: not significant, *P<0.05, ***P*<0.01, ***P < 0.001, *****P* < 0.0001.

**Figure S5. Lymphoid lineage analysis in the peripheral blood and spleen of NLRP3 gene knockout mice.**

(A) Flow cytometry analysis of the percentage of T and B lymphocytes in the peripheral blood of wild-type (WT) and Caspase-3^-/-^ (KO) mice (n=3). (B) Flow cytometry analysis of the percentage of T and B lymphocytes in the spleen of WT and Caspase-3 KO mice (n=3). All data are presented as mean±SEM. ns: not significant, **P*<0.05, ***P*<0.01.

**Figure S6. SCF and CXCL12 level determination in MSC subpopulations.**

(A-B) Sca1^+^ BA-MSCs were isolated from WT mice, sorted using magnetic beads, and cultured in MEM-alpha medium for 9 days. The gene expression of NLRP3 (A) and the secretion of SCF and CXCL12 in the supernatants (B) were examined respectively at different time points (n=3-4). (C) Flow cytometry plots and analysis of the frequency of Sca1^+^, CD146^+^ and CD166^+^ BA-MSCs in NLRP3 KO mice (n=3). All data are presented as mean±SEM. ns: not significant, **P*<0.05, ***P*<0.01, ****P* < 0.001, *****P* < 0.0001.

**Table S1. Primer sequences in quantitative real-time PCR.**

| Gene | Primer(5’-3’) |
| --- | --- |
| SCF-F | GAATCTCCGAAGAGGCCAGAA |
| SCF-R | GCTGCAACAGGGGGTAACAT |
| CXCL12-F | TGCATCAGTGACGGTAAACCA |
| CXCL12-R | TTCTTCAGCCGTGCAACAATC |
| G-CSF-F | ATGGCTCAACTTTCTGCCCAG |
| G-CSF-R | CTGACAGTGACCAGGGGAAC |
| M-CSF-F | GGCTTGGCTTGGGATGATTCT |
| M-CSF-R | GAGGGTCTGGCAGGTACTC |
| GM-CSF-F | GGCCTTGGAAGCATGTAGAGG |
| GM-CSF-R | GGAGAACTCGTTAGAGACGACTT |
| IL-6-F | TAGTCCTTCCTACCCCAATTTCC |
| IL-6-R | TTGGTCCTTAGCCACTCCTTC |
| NLRP3-F | ATTACCCGCCCGAGAAAGG |
| NLRP3-R | TCGCAGCAAAGATCCACACAG |
| GAPDH-F | AGGTCGGTGTGAACGGATTTG |
| GAPDH-R | TGTAGACCATGTAGTTGAGGTCA |
