# Supplementary figures and images for: Caspase-3/NLRP3 signaling in the mesenchymal stromal niche regulates myeloid-biased hematopoiesis
Source: Stem Cell Res Ther. 2021 Nov 20;12:579. doi: 10.1186/s13287-021-02640-y (PMC8605603; doi:10.1186/s13287-021-02640-y)

## Slide 1
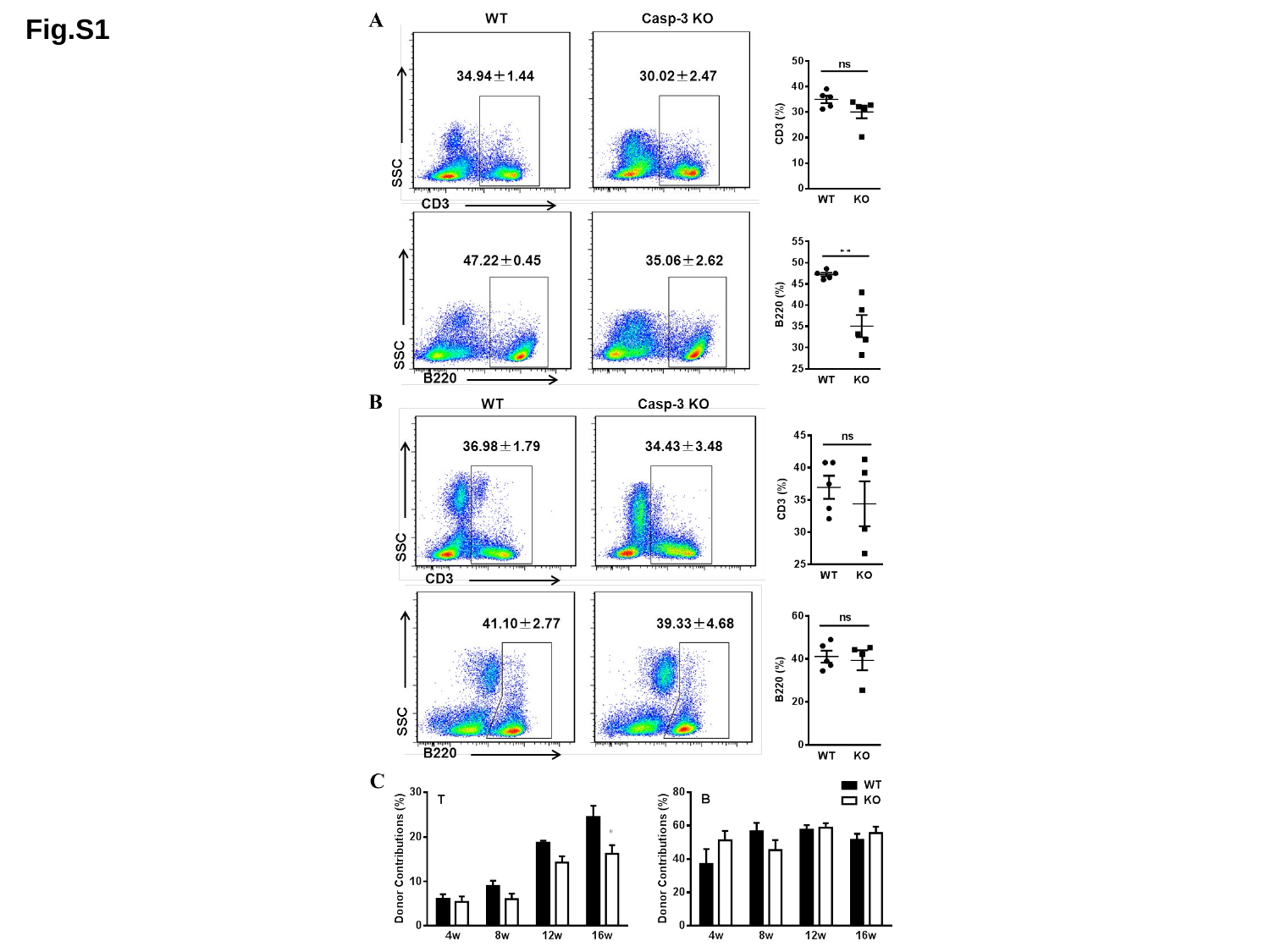

Fig.S1

## Slide 2
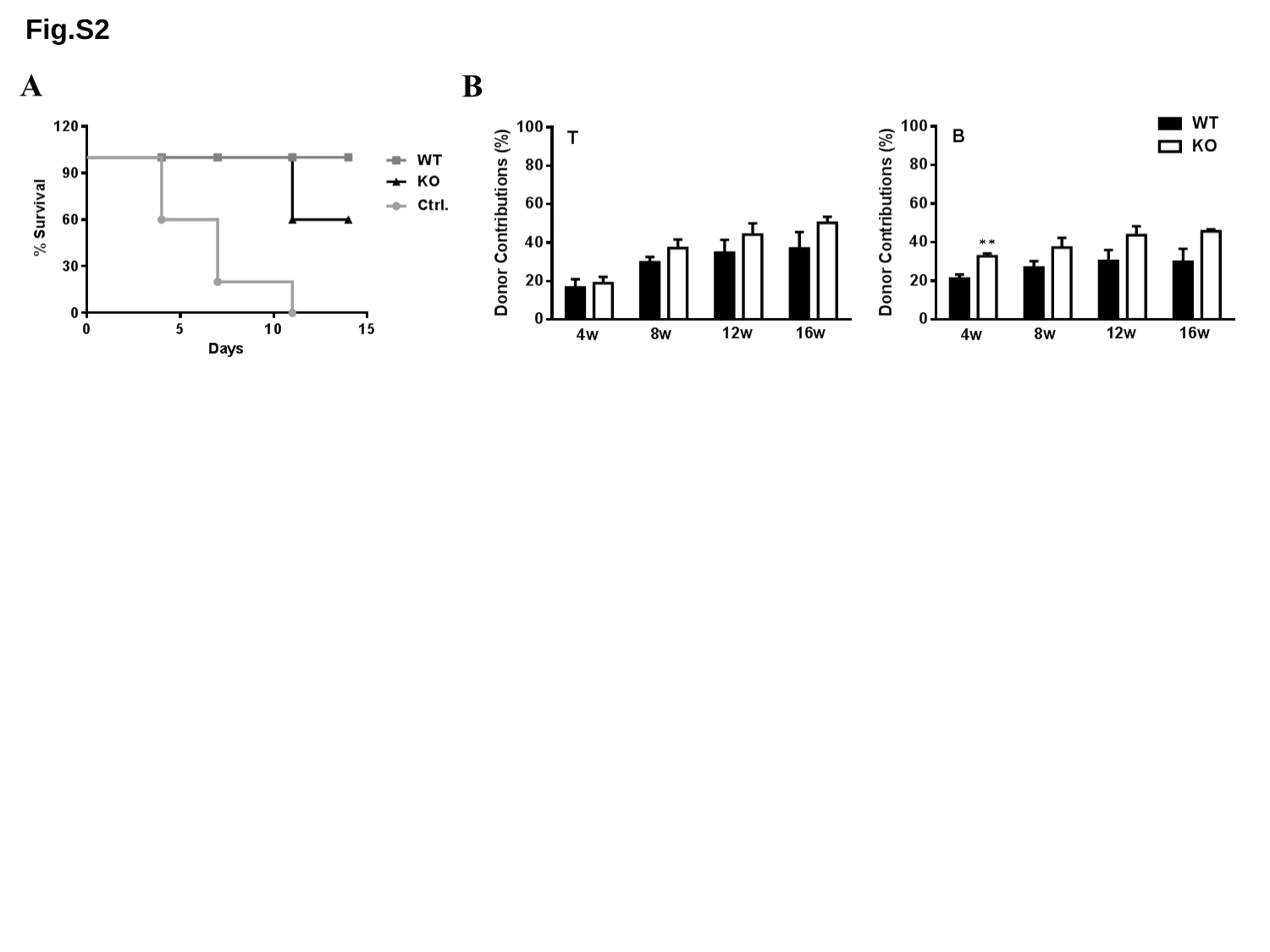

Fig.S2

## Slide 3
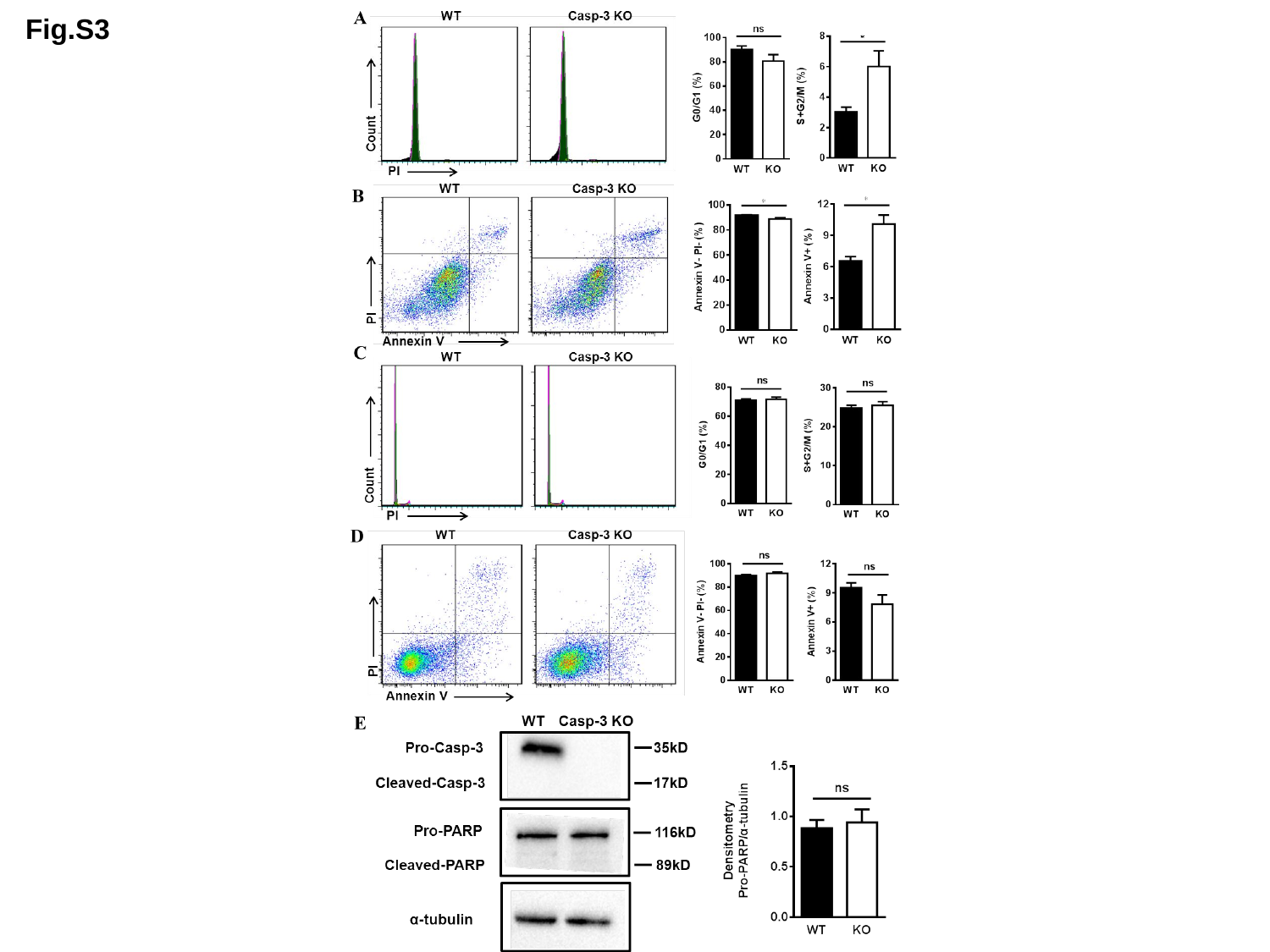

Fig.S3

## Slide 4
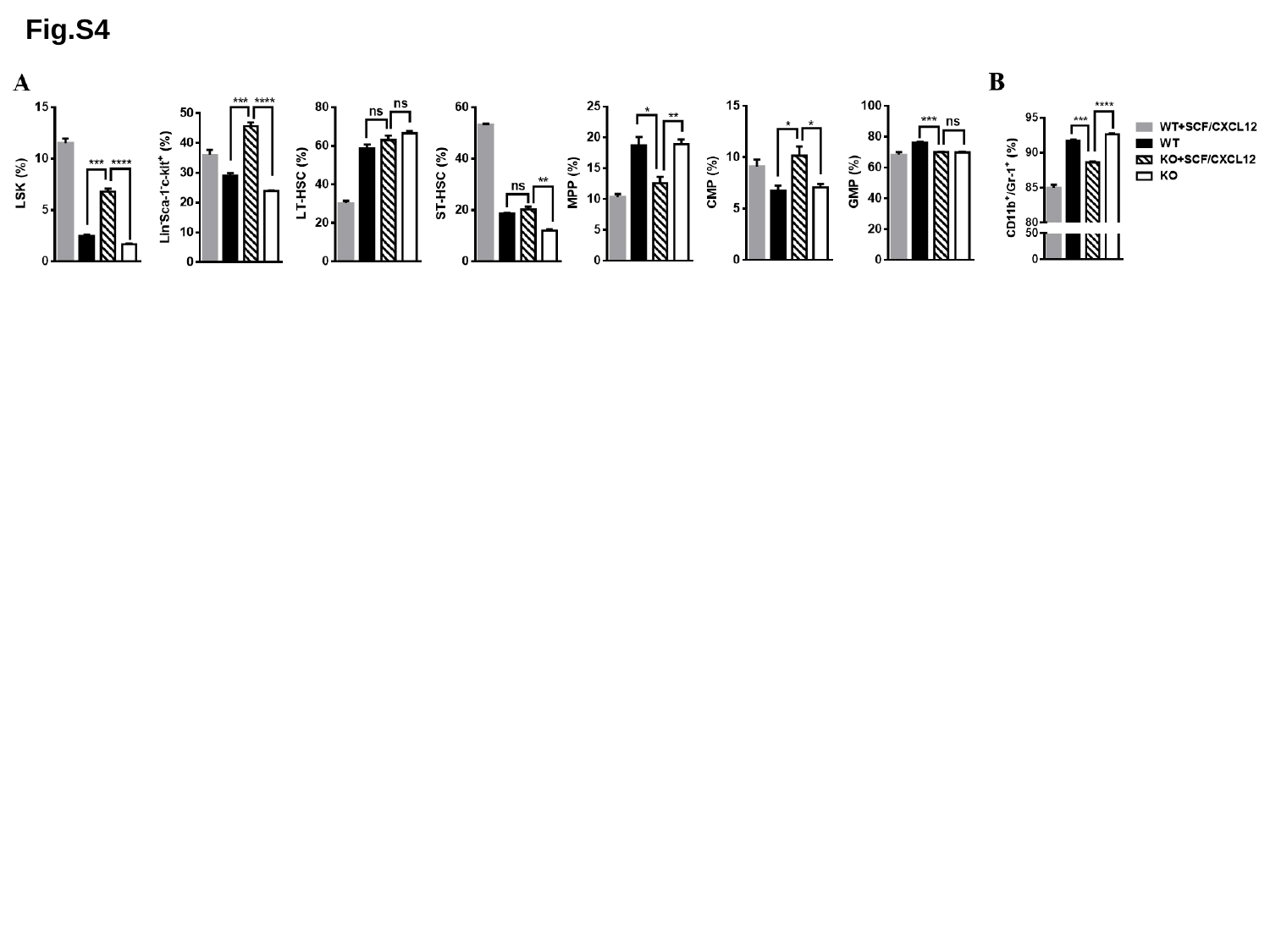

Fig.S4

## Slide 5
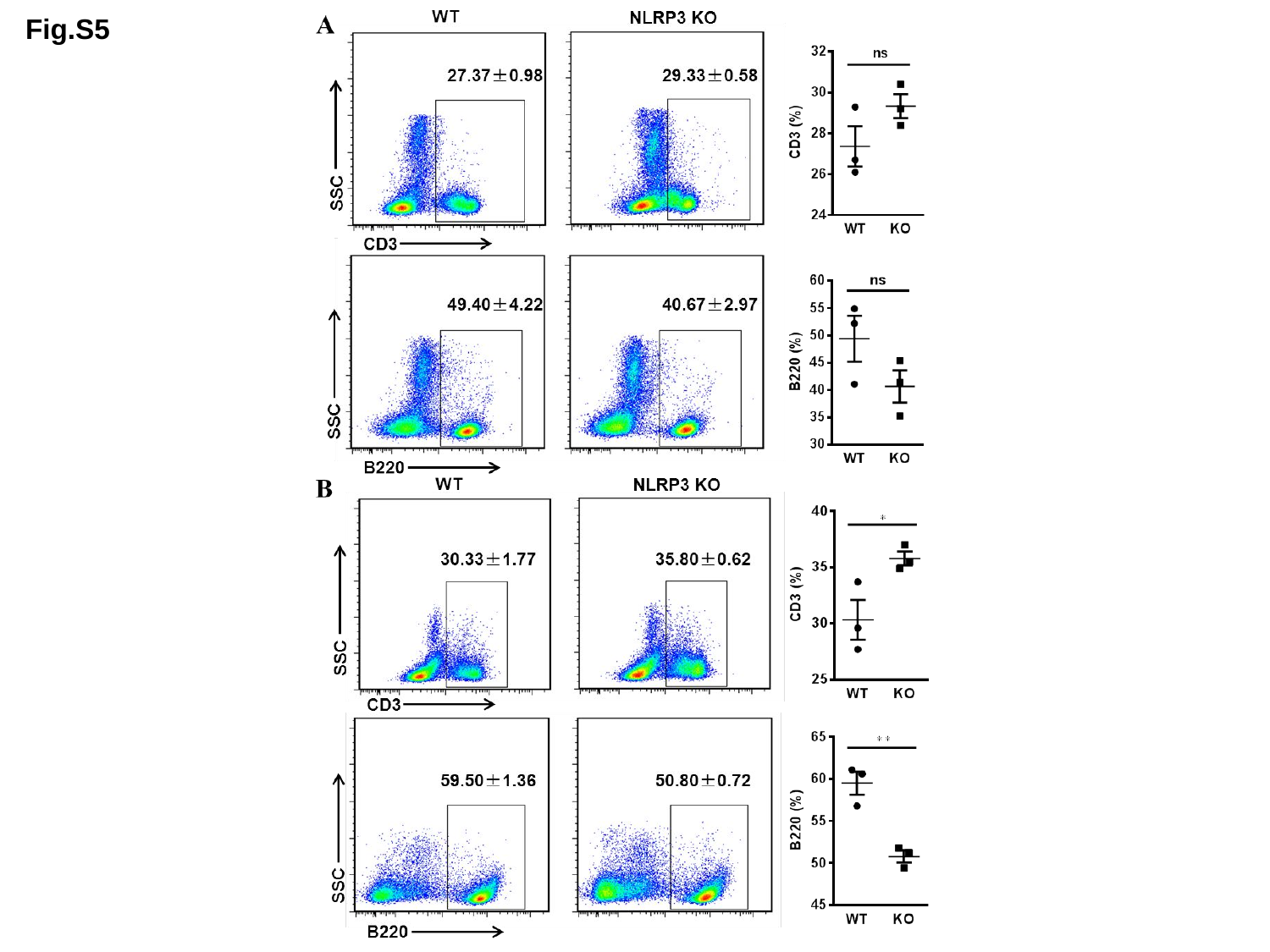

Fig.S5

## Slide 6
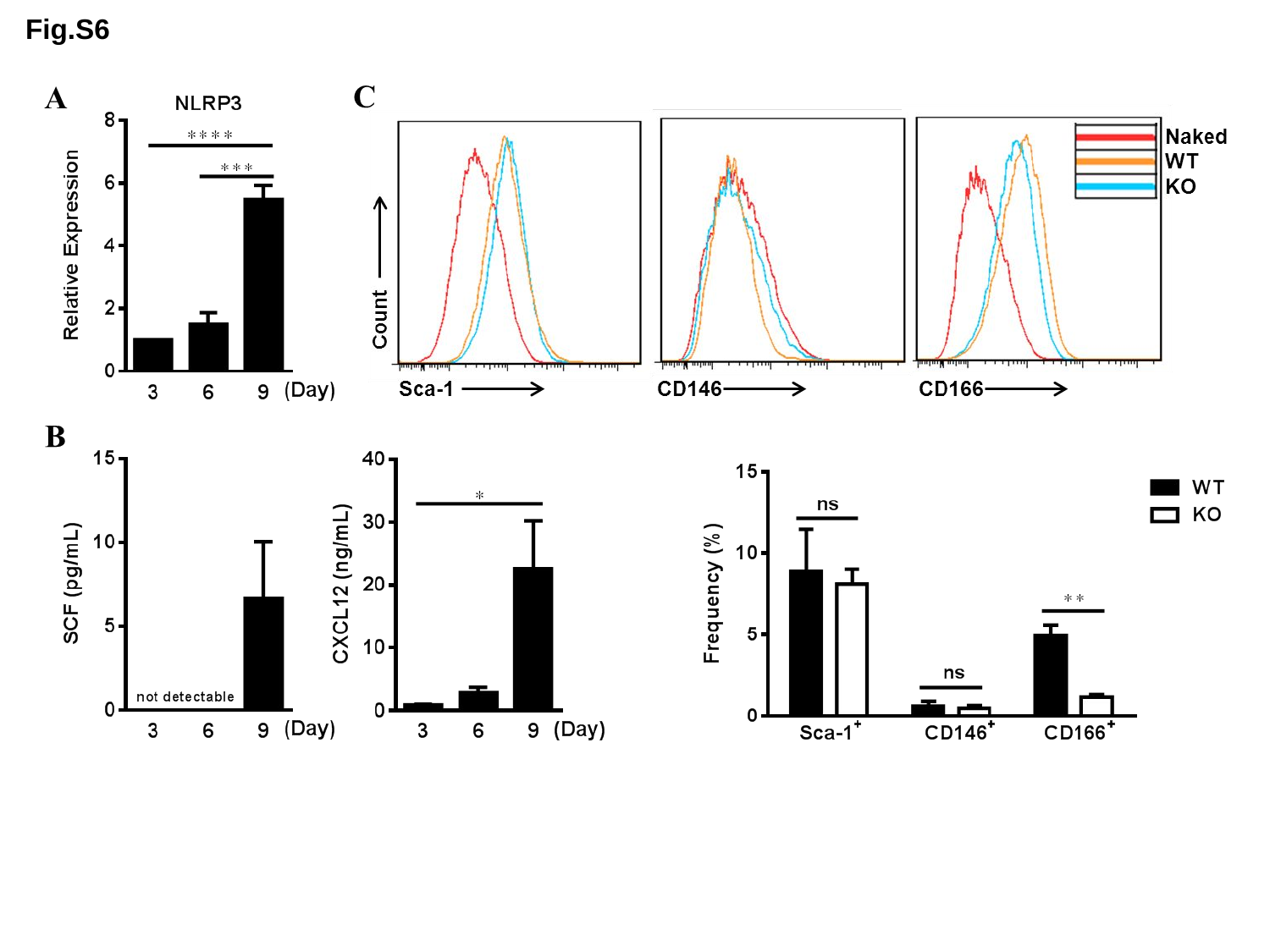

Fig.S6

Supplement: Supplementary file 2 — Additional file 2: Supplementary figures. [file 13287_2021_2640_MOESM2_ESM.ppt]
